# Supplementary material for: A Simple Method of Synthesis of 3-Carboxy-2,2,5,5-Tetraethylpyrrolidine-1-oxyl and Preparation of Reduction-Resistant Spin Labels and Probes of Pyrrolidine Series
Source: Molecules. 2021 Sep 23;26(19):5761. doi: 10.3390/molecules26195761 (PMC8510269; doi:10.3390/molecules26195761)

A simple method of synthesis of 3-carboxy-2,2,5,5-tetraethylpyrrolidine-1-oxyl and preparation of reduction-resistant spin labels and probes of pyrrolidine series

Sergey A. Dobrynin,[1] Mikhail S. Usatov,[1,2] Irina F. Zhurko,[1] Denis A. Morozov,[1] Yuliya F. Polienko,[1] Yurii I. Glazachev,[3] Dmitriy A. Parkhomenko,[1] Mikhail A. Tyumentsev,[4] Yuri V. Gatilov,[1] Elena I. Chernyak,[1] Elena G. Bagryanskaya,[1] Igor A. Kirilyuk[1]

[1]N. N. Vorozhtsov Novosibirsk Institute of Organic Chemistry SB RAS, Lavrentiev Ave. 9, Novosibirsk, 630090, Russia; dobrynin@nioch.nsc.ru

[2]Novosibirsk State University, Pirogova Str. 2, Novosibirsk, 630090, Russia

[3]Voevodsky Institute of Chemical Kinetics and Combustion SB RAS, Institutskaya 3, Novosibirsk, 630090, Russia

[4]Federal Research Center Institute of Cytology and Genetics SB RAS, Lavrentiev Ave. 10,Novosibirsk, 630090, Russia

Supporting Information

Table of contents

NMR spectra S4

^{1}H NMR (400 MHz; $CD_3OD/CDCl_3$, Zn/CF_3COOH) of 3-carboxy-2,2,5,5-tetraethylpyrrolidine-1-oxyl (1). S4

^{1}H NMR (400 MHz; $CDCl_3$) of 3-carboxy-2,2,5-triethyl-3,4-dihydro-2*H*-pyrrole 1-oxide (8). . S4

^{13}C{^{1}H} NMR (100 MHz; $CDCl_3$) of 3-carboxy-2,2,5-triethyl-3,4-dihydro-2*H*-pyrrole 1-oxide (8). S5

^{1}H NMR (400 MHz; $CD_3OD/CDCl_3$, Zn/CF_3COOH) of 3-carboxy-5-ethynyl-2,2,5-triethylpyrrolidine-1-oxyl – mixture of isomers (10a,b). S5

^{1}H NMR(400 MHz; $CD_3OD/CDCl_3$, Zn/CF_3COOH) of 3-carboxy-5-ethynyl-2,2,5-triethylpyrrolidine-1-oxyl –major isomer (10a). S6

^{1}H NMR(400 MHz; $CD_3OD/CDCl_3$, Zn/CF_3COOH) of 3-carboxy-5-vinyl-2,2,5-triethylpyrrolidine-1-oxyl (14). S6

^{1}H NMR (400 MHz; $CD_3OD/CDCl_3$) of 3-carboxy-2,2,5,5-tetraethylpyrrolidine (15)........ S7

^{13}C{^{1}H} NMR (100 MHz; $CD_3OD/CDCl_3$) of 3-carboxy-2,2,5,5-tetraethylpyrrolidine (15). S7

^{1}H NMR (100 MHz;$CDCl_3$) of 1-hydroxy-3-methoxycarbonyl-2,2,5,5-tetraethylpyrrolidine hydrochloride (17a,b) S8

^{13}C{^{1}H} NMR (75 MHz; $CDCl_3$) of 1-Hydroxy-3-methoxycarbonyl-2,2,5,5-tetraethylpyrrolidine hydrochloride (17a,b) S9

^{1}H NMR (300 MHz; $CD_3OD/CDCl_3$, Zn/CF_3COOH) of 3-hydroxymethyl -2,2,5,5-tetraethylpyrrolidine-1-oxyl (18). S9

^{1}H NMR (500 MHz; $CD_3OD/CDCl_3$, Zn/CF_3COOH) of 3-hydroxymethyl-5-ethynyl-2,2,5-triethylpyrrolidine-1-oxyl mixture of isomers (19a,b). S10

^{1}H NMR (300 MHz; $CD_3OD/CDCl_3$, Zn/CF_3COOH) of 3-methansulfonyloxy-methyl-2,2,5,5-tetraethylpyrrolidine-1-oxyl (20). S11

^{1}H NMR (300 MHz; $CD_3OD/CDCl_3$, Zn/CF_3COOH) of 3-aminomethyl -2,2,5,5-tetraethylpyrrolidine-1-oxyl (22). S11

^{1}H NMR (300 MHz; CD3OD/CDCl3, Zn/CF3COOH) of 3-(2-triphenylphosphonio-acetamido)methyl-2,2,5,5-tetraethylpyrrolidine-1-oxyl chloride (28). S12

^{1}HNMR (300 MHz; $CD_3OD/CDCl_3$, Zn/CF_3COOH) of 3-(2-(pyrrolidin-1yl)acetamidomethyl)-2,2,5,5-tetraethylpyrrolidine-1-oxyl (29) S12

^{1}HNMR (300 MHz; $CD_3OD/CDCl_3$, Zn/CF_3COOH) of 3-((piperidin-1-yl)methyl)-2,2,5,5-tetraethylpyrrolidine-1-oxyl (30). S13

^{1}H NMR(300 MHz; $CD_3OD/CDCl_3$, Zn/CF_3COOH) of 3-(dimethylaminomethyl)-2,2,5,5-tetraethylpyrrolidine-1-oxyl (31). S13

Line shape analysis of multiplets for mixture of isomers 10a,b. S14

Fig. S1 Simulation of spin system for ^{1}H NMR (400 MHz; $CD_3OD/CDCl_3$, Zn/CF_3COOH system) of 3-carboxy-5-ethinyl-2,2,5-triethylpyrrolidine-1-oxyl – mixture of isomers (10a,b). S14

Table S1.Simulation of spin system parameters for ^{1}H NMR (400 MHz; $CD_3OD/CDCl_3$, Zn/CF_3COOH system) of 3-carboxy-5-ethinyl-2,2,5-triethylpyrrolidine-1-oxyl – mixture of isomers (10a,b). S14

IR spectra S15

IR (KBr) of 3-carboxy-2,2,5,5-tetraethylpyrrolidine-1-oxyl (1). S15

IR (neat) of 3-[[(2,5-dioxo-1-pyrrolidinyl)oxy]carbonyl]-2,2,5,5-tetraethylpyrrolidine-1-oxyl (3). S15

IR (KBr) of 3-carboxy-2,2,5-triethyl-3,4-dihydro-2H-pyrrole 1-oxide (8). S15

IR (KBr) of 3-carboxy-5-ethynyl-2,2,5-triethylpyrrolidine-1-oxyl (10a,b). S16

IR (KBr) of 3-carboxy-5-vinyl-2,2,5-triethylpyrrolidine-1-oxyl (14). S16

IR (KBr) of 3-carboxy-2,2,5,5-tetraethylpyrrolidine (15). S16

IR (neat) of 3-methoxycarbonyl-2,2,5,5-tetraethylpyrrolidine-1-oxyl (16). S17

IR (KBr) of 1-hydroxy-3-methoxycarbonyl-2,2,5,5-tetraethylpyrrolidine hydrochloride (17) . S17

IR (neat) of 3-hydroxymethyl-2,2,5,5-tetraethylpyrrolidine-1-oxyl (18). S17

IR (neat) of 3-hydroxymethyl-5-ethinyl-2,2,5-triethylpyrrolidine-1-oxyl (19a,b). S18

IR (neat) of 3-brommethyl-2,2,5,5-tetraethylpyrrolidine-1-oxyl (20a). S18

IR (neat) of 3-mesyloxymethyl-2,2,5,5-tetraethylpyrrolidine-1-oxyl (20b). S18

IR (neat) of 3-azidomethyl-2,2,5,5-tetraethylpyrrolidine-1-oxyl (21). S19

IR (neat) of 3-aminomethyl-2,2,5,5-tetraethylpyrrolidine-1-oxyl (22). S19

IR (KBr) of 3-maleimidomethyl-2,2,5,5-tetraethylpyrrolidine-1-oxyl (24). S19

IR (neat) of 3-bromoacetamidomethyl-2,2,5,5-tetraethylpyrrolidine-1-oxyl (25). S20
IR (neat) of 3-Chloroacetamidomethyl-2,2,5,5-tetraethylpyrrolidine-1-oxyl (26)..................... S20
IR (KBr) of 3-Iodoacetamidomethyl-2,2,5,5-tetraethylpyrrolidine-1-oxyl (27). S20
IR (KBr) of 3-(2-triphenylphosphonio-acetamido)methyl-2,2,5,5-tetraethylpyrrolidine-1-oxyl chloride (28). S21
IR (neat) of 3-(2-(pyrrolidin-1yl)acetamidomethyl)-2,2,5,5-tetraethylpyrrolidine-1-oxyl (29). S21
IR (neat) of 3-((piperidin-1-yl)methyl)-2,2,5,5-tetraethylpyrrolidine-1-oxyl (30)..................... S21
IR (neat) of 3-(dimethylaminomethyl)-2,2,5,5-tetraethylpyrrolidine-1-oxyl (31). S22
HPLC analyses S23
HPLC of 3-carboxy-5-ethinyl-2,2,5-triethylpyrrolidine-1-oxyl major isomer(19a). S23
HPLC of 3-((piperidin-1-yl)methyl)-2,2,5,5-tetraethylpyrrolidine-1-oxyl (30)......................... S24
HPLC of 3-(2-(pyrrolidin-1yl)acetamidomethyl)-2,2,5,5-tetraethylpyrrolidine-1-oxyl (29)..... S25
Table S2. X-ray experimental details S26
Fig.S2. X-Ray of 3-carboxy-5-ethynyl-2,2,5-triethylpyrrolidine-1-oxyl (10a)......................... S27
Fig. S3. The dependences of the first order rates of radical reductions versus ascorbic acid concentrations. S28

NMR spectra

^{1}H NMR (400 MHz; $CD_3OD/CDCl_3$, Zn/CF_3COOH) of 3-carboxy-2,2,5,5-tetraethylpyrrolidine-1-oxyl (1).

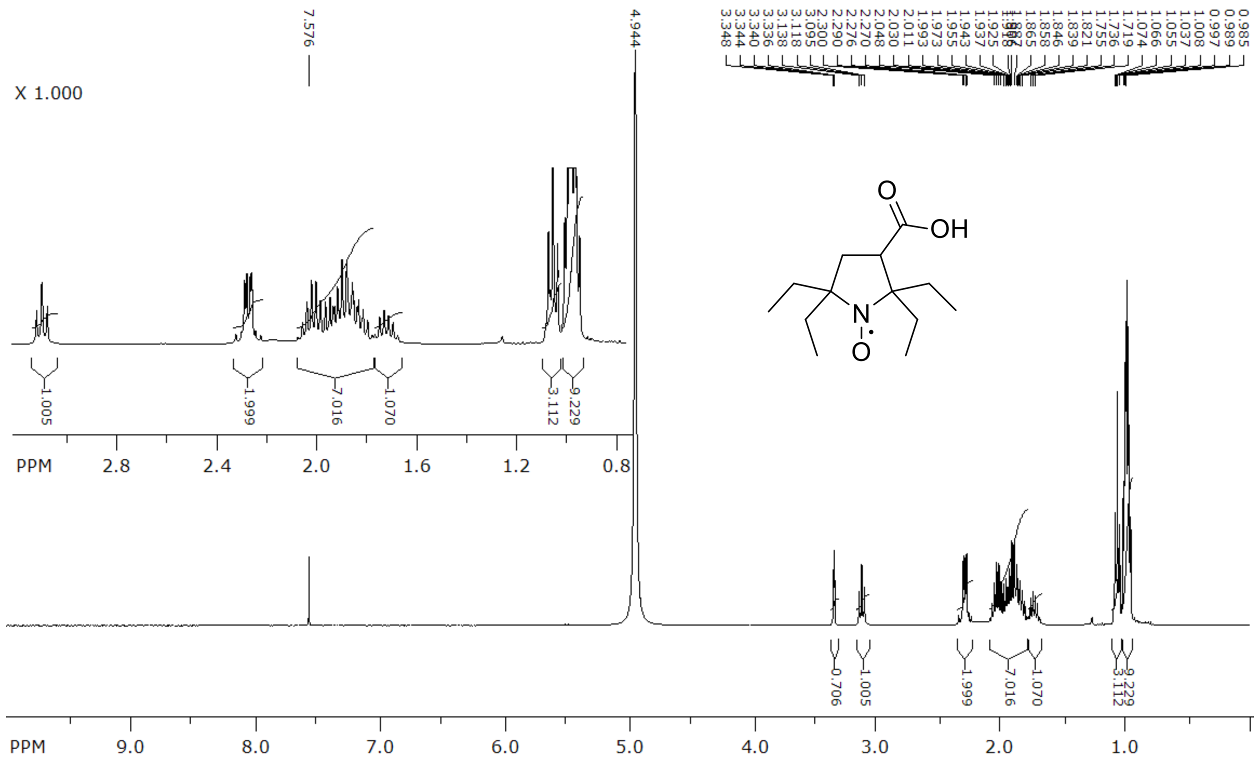

^{1}H NMR (400 MHz; $CDCl_3$) of 3-carboxy-2,2,5-triethyl-3,4-dihydro-2*H*-pyrrole 1-oxide (8).

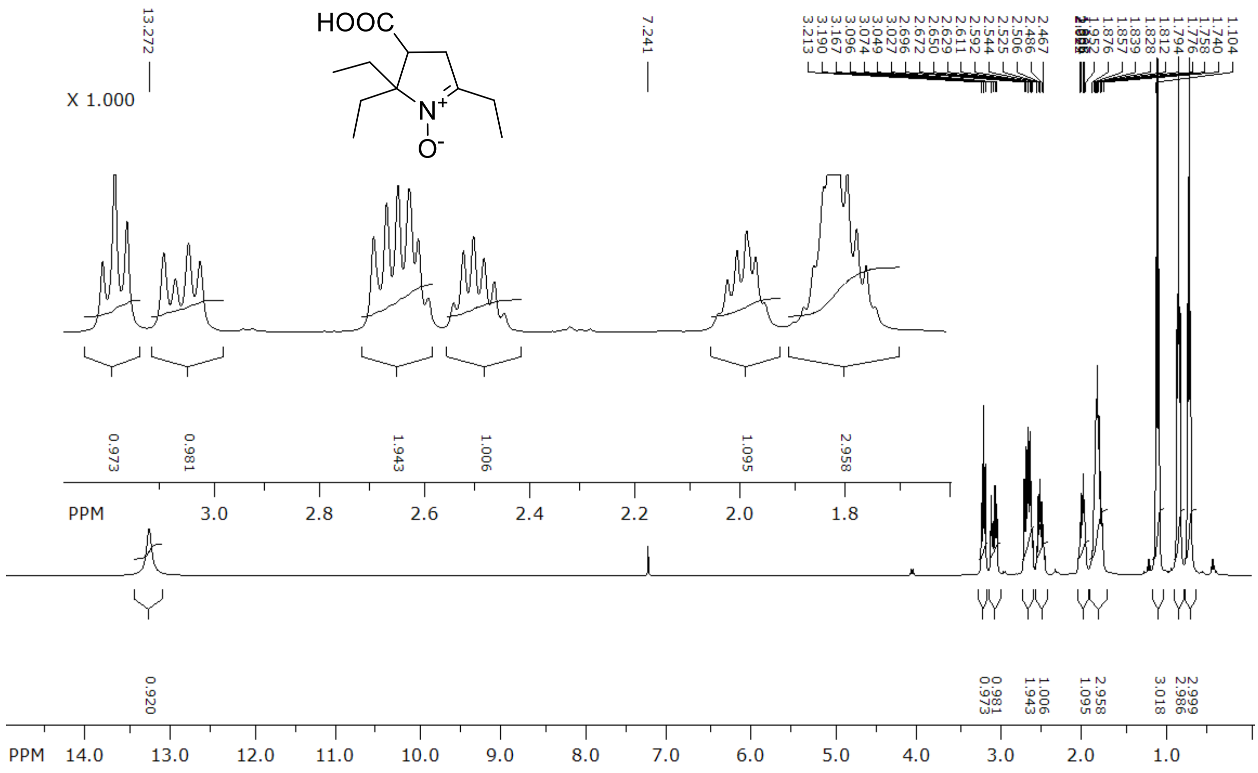

$^{13}C\{^{1}H\}$ NMR (100 MHz; $CDCl_3$) of 3-carboxy-2,2,5-triethyl-3,4-dihydro-2*H*-pyrrole 1-oxide (8).

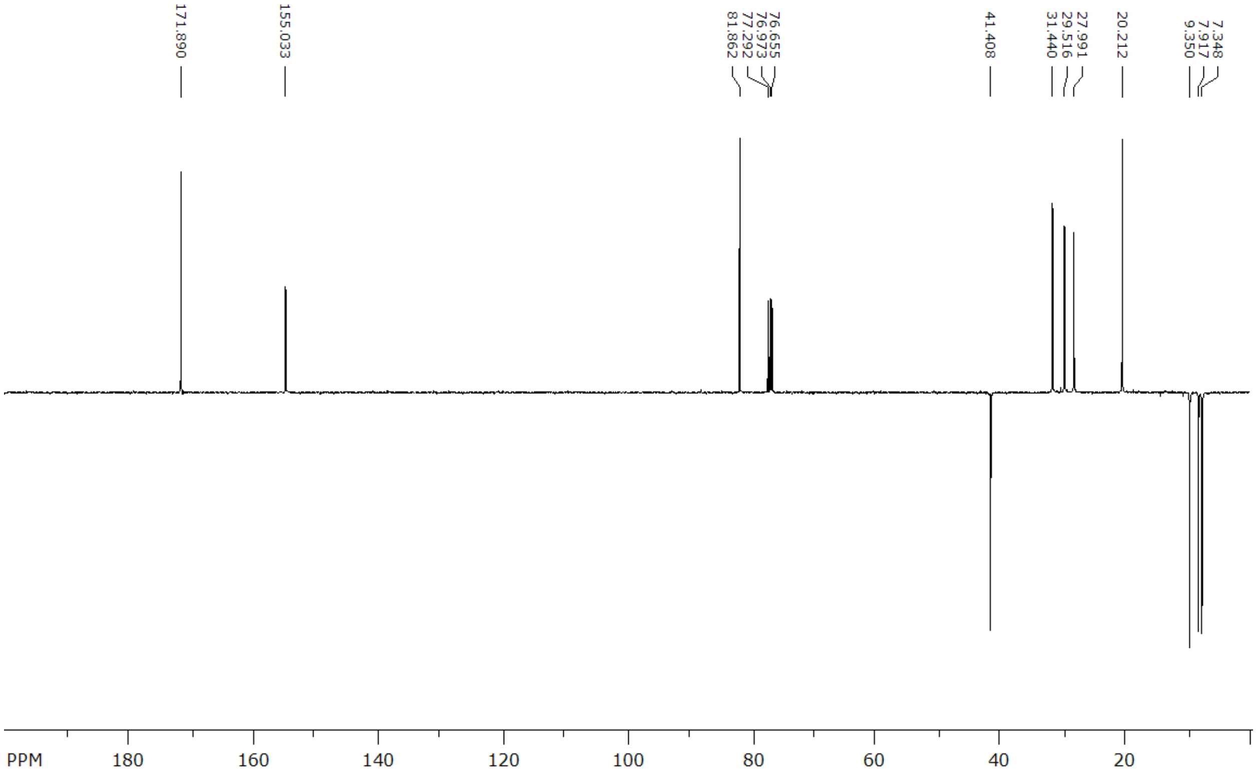

^{1}H NMR (400 MHz; $CD_3OD/CDCl_3$, Zn/CF_3COOH) of 3-carboxy-5-ethynyl-2,2,5-triethylpyrrolidine-1-oxyl – mixture of isomers (10a,b).

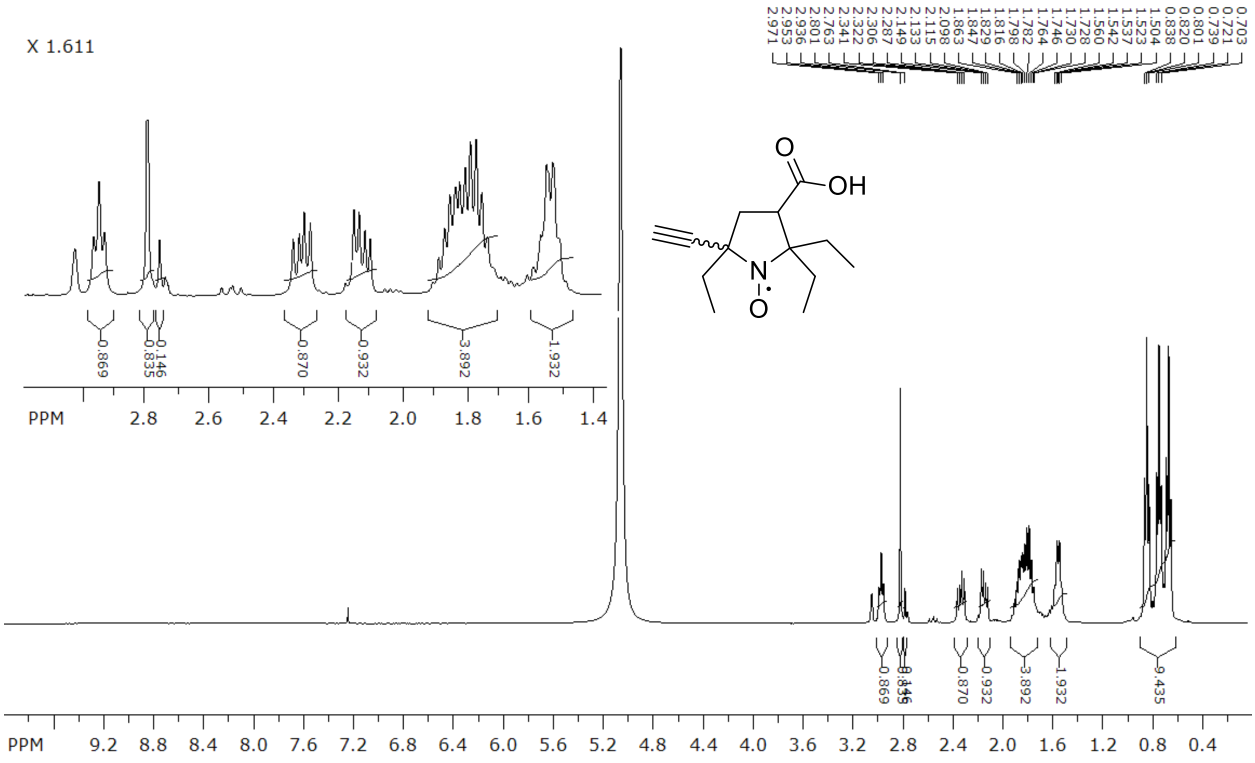

^1H NMR(400 MHz; $CD_3OD/CDCl_3$, Zn/CF_3COOH) of 3-carboxy-5-ethynyl-2,2,5-triethylpyrrolidine-1-oxyl –major isomer (10a).

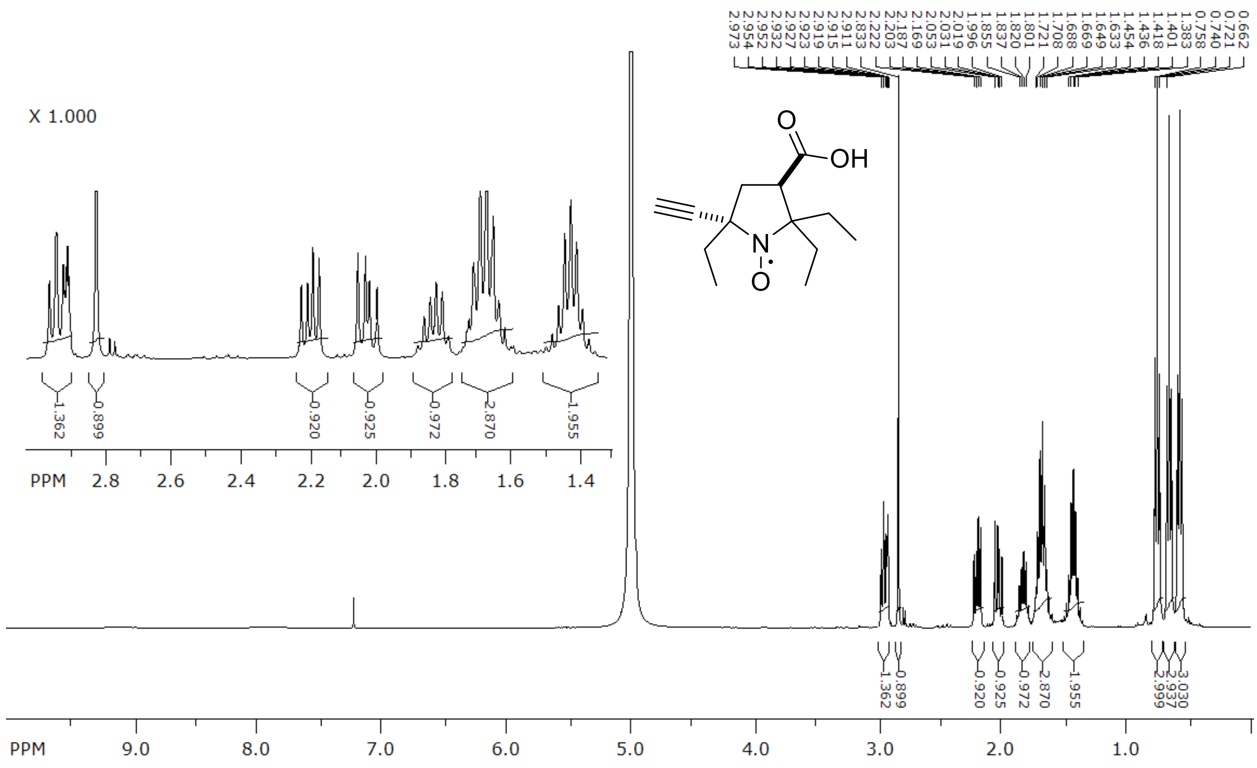

^1H NMR(400 MHz; $CD_3OD/CDCl_3$, Zn/CF_3COOH) of 3-carboxy-5-vinyl-2,2,5-triethylpyrrolidine-1-oxyl (14).

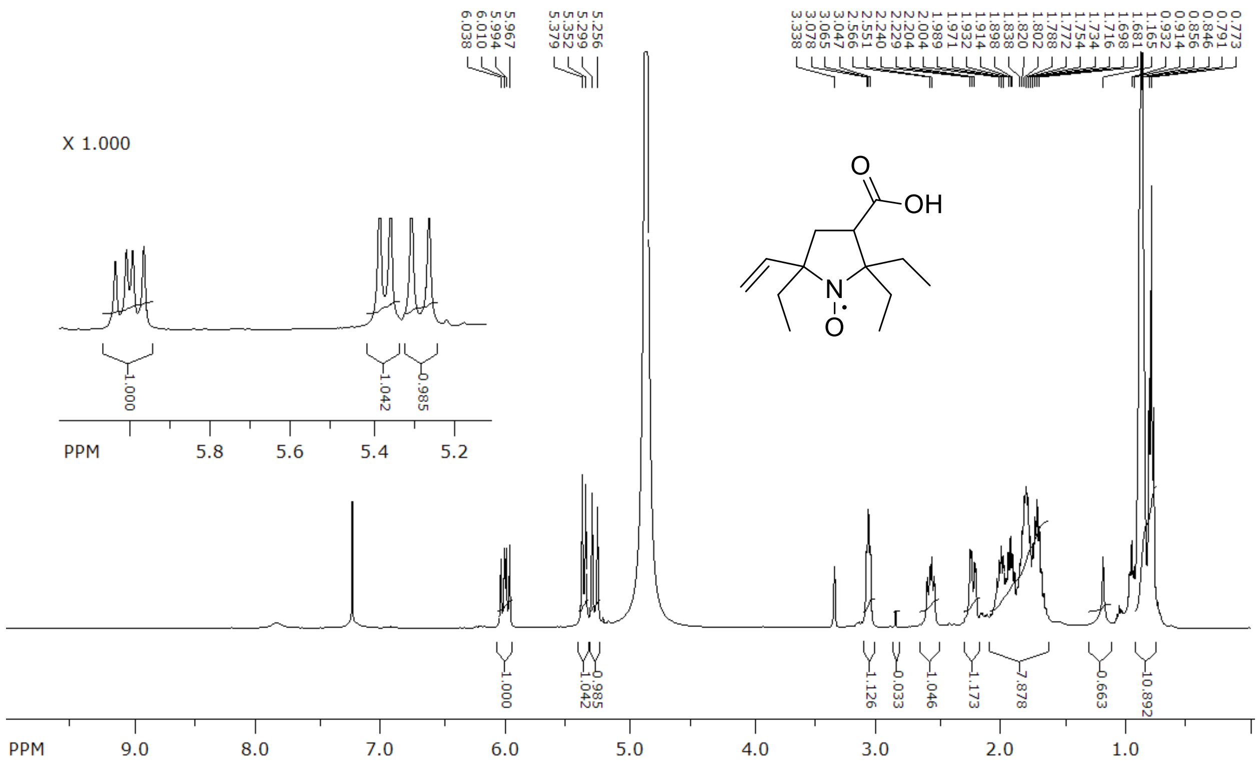

^{1}H NMR (400 MHz; $CD_3OD/CDCl_3$) of 3-carboxy-2,2,5,5-tetraethylpyrrolidine (15).

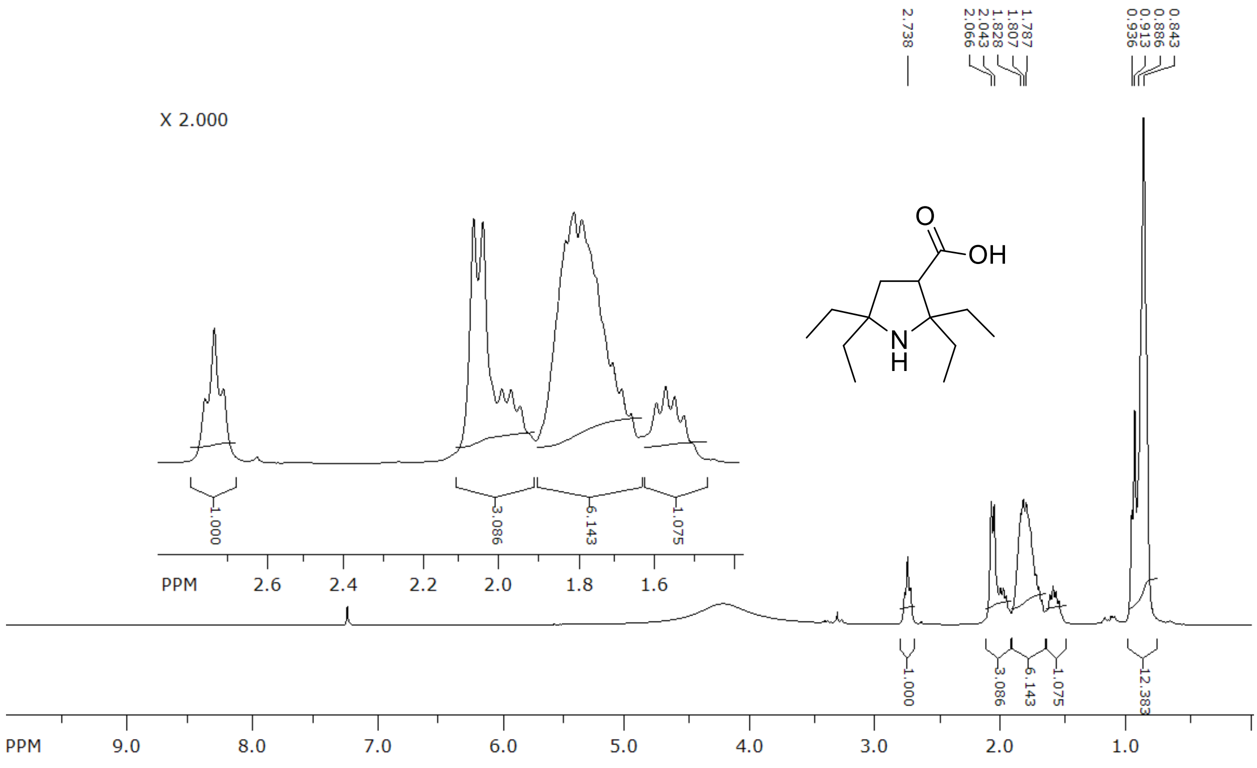

$^{13}C\{^{1}H\}$ NMR (100 MHz; $CD_3OD/CDCl_3$) of 3-carboxy-2,2,5,5-tetraethylpyrrolidine (15).

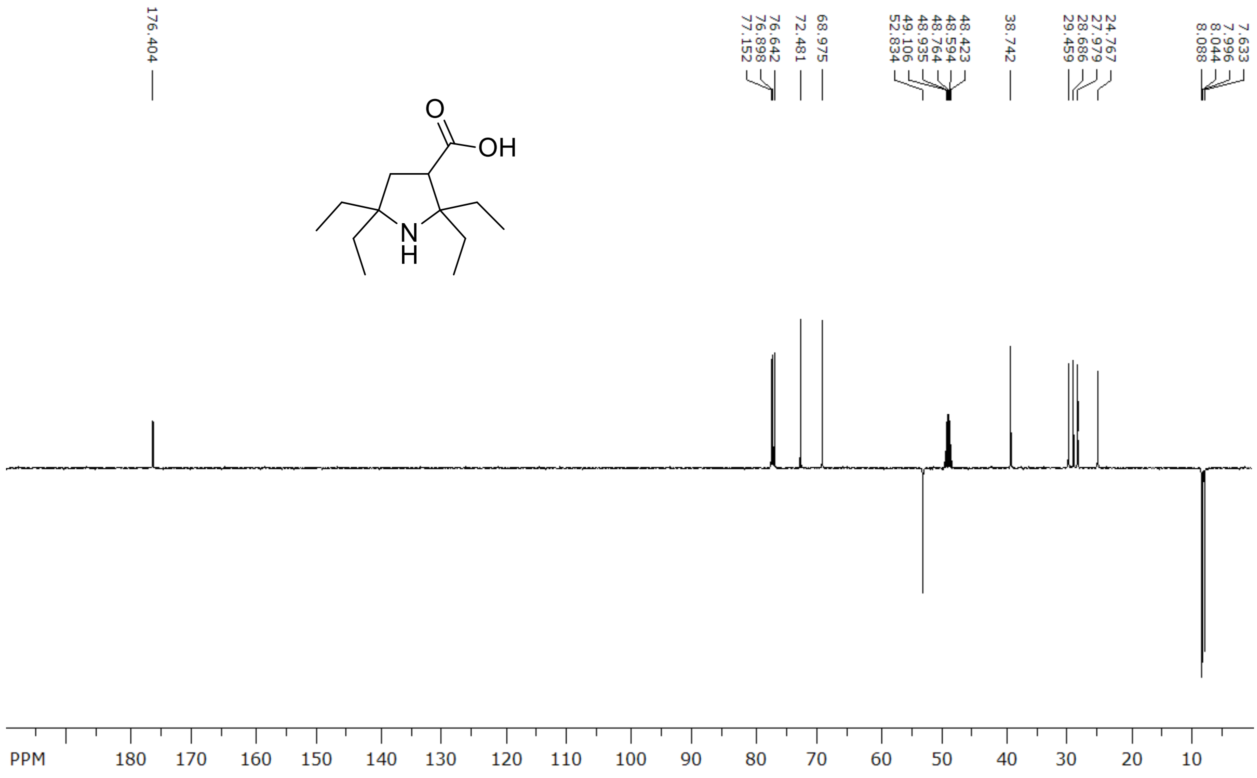

^{1}H NMR (100 MHz;$CDCl_3$) of 1-hydroxy-3-methoxycarbonyl-2,2,5,5-tetraethylpyrrolidine hydrochloride (17a,b)

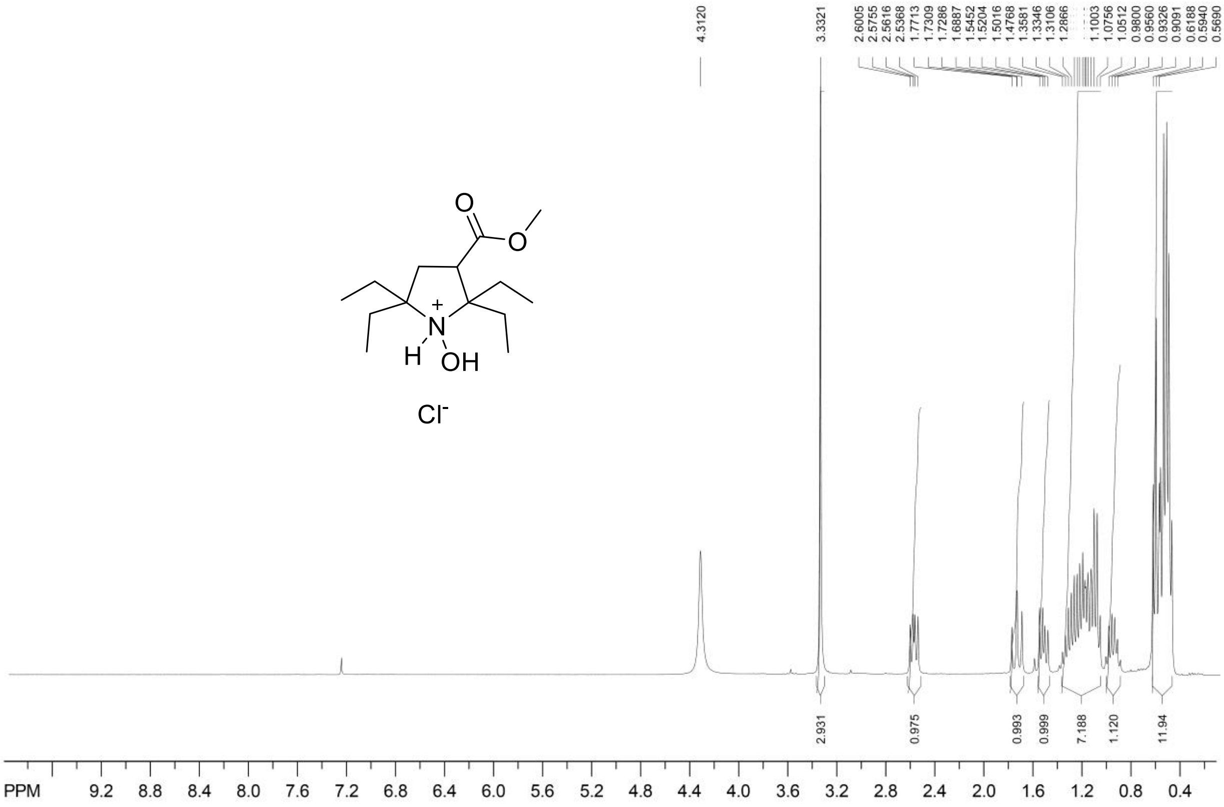

$^{13}C\{^1H\}$ NMR (75 MHz; $CDCl_3$) of 1-Hydroxy-3-methoxycarbonyl-2,2,5,5-tetraethylpyrrolidine hydrochloride (17a,b)

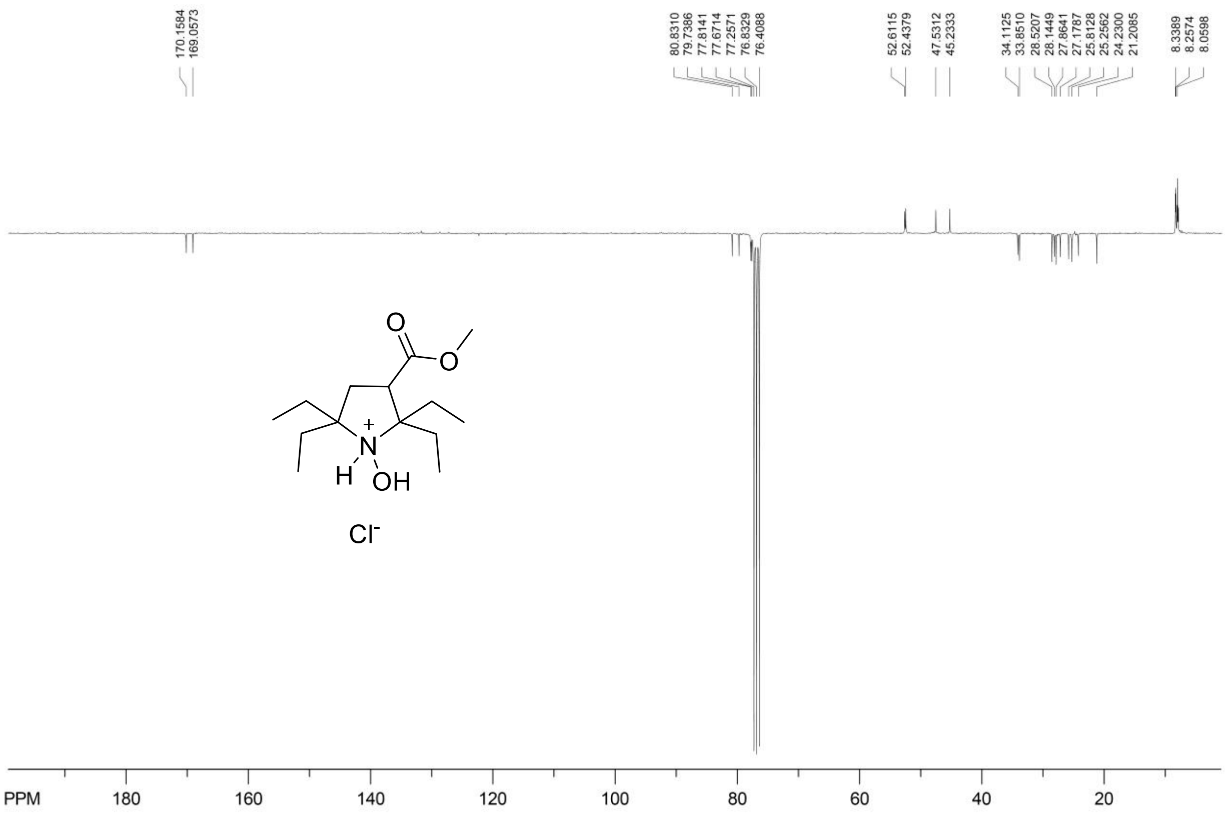

^1H NMR (300 MHz; $CD_3OD/CDCl_3$, Zn/CF_3COOH) of 3-hydroxymethyl -2,2,5,5-tetraethylpyrrolidine-1-oxyl (18).

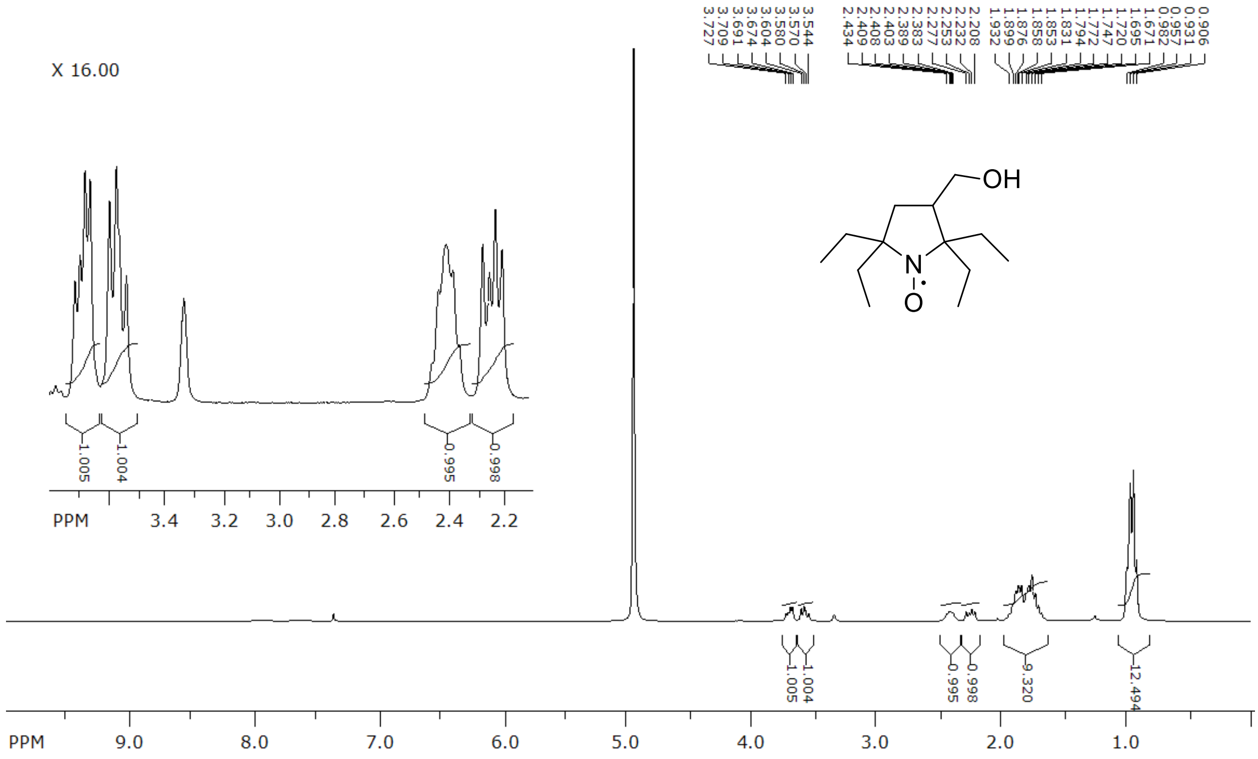

^{1}H NMR (500 MHz; $CD_3OD/CDCl_3$, Zn/CF_3COOH) of 3-hydroxymethyl-5-ethynyl-2,2,5-triethylpyrrolidine-1-oxyl mixture of isomers (19a,b).

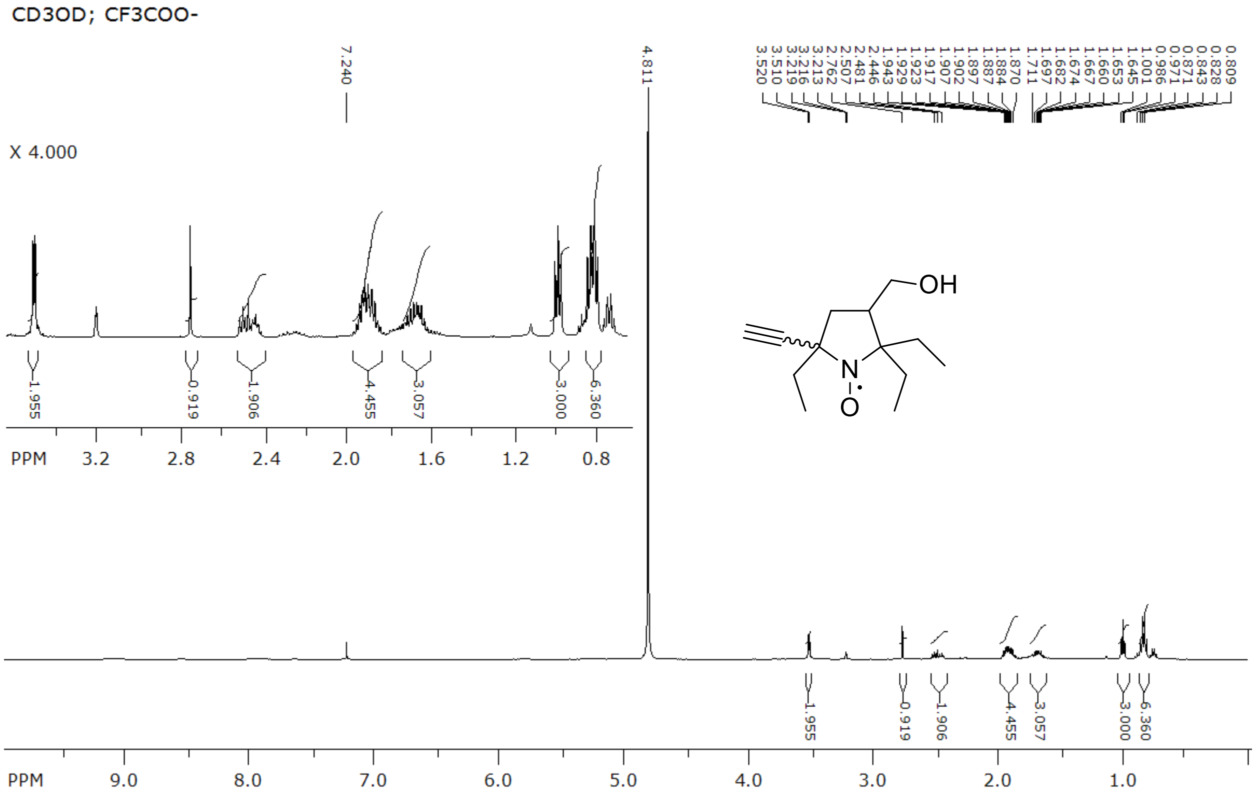

^{1}H NMR (300 MHz; $CD_3OD/CDCl_3$, Zn/CF_3COOH) of 3-methansulfonyloxy-methyl-2,2,5,5-tetraethylpyrrolidine-1-oxyl (20).

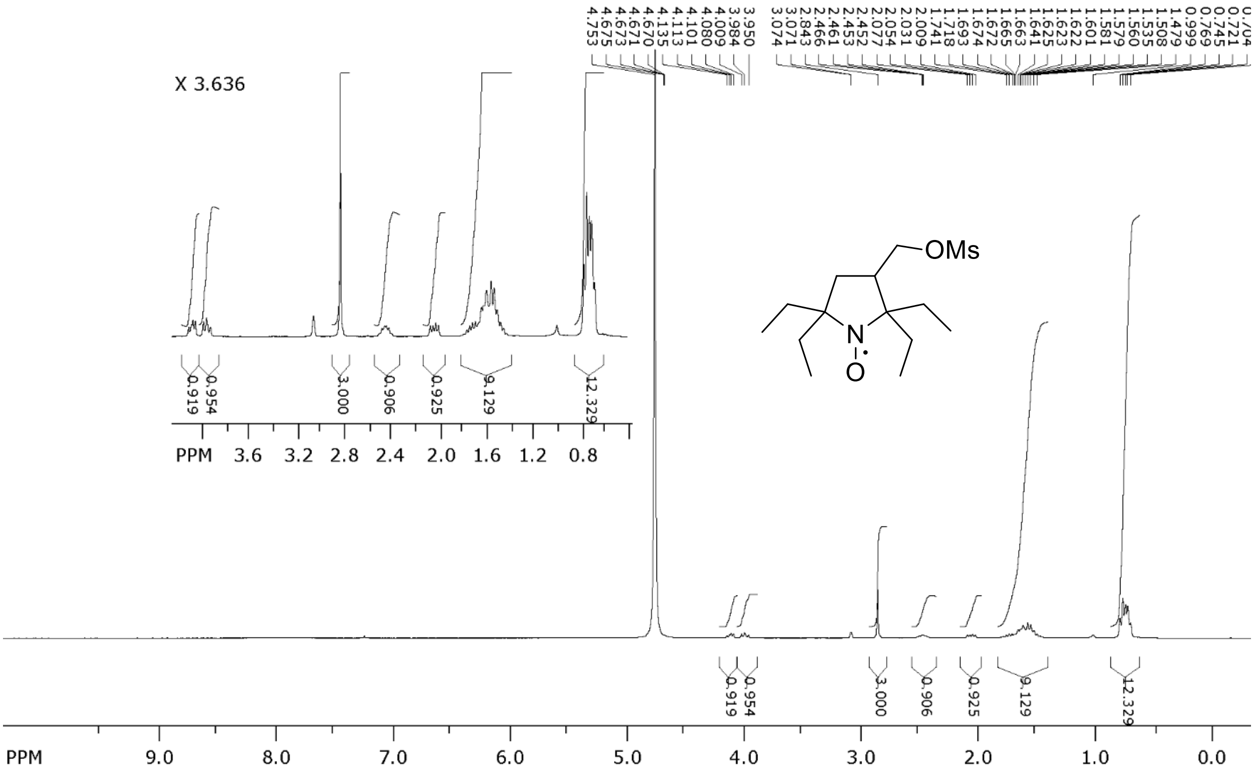

^{1}H NMR (300 MHz; $CD_3OD/CDCl_3$, Zn/CF_3COOH) of 3-aminomethyl -2,2,5,5-tetraethylpyrrolidine-1-oxyl (22).

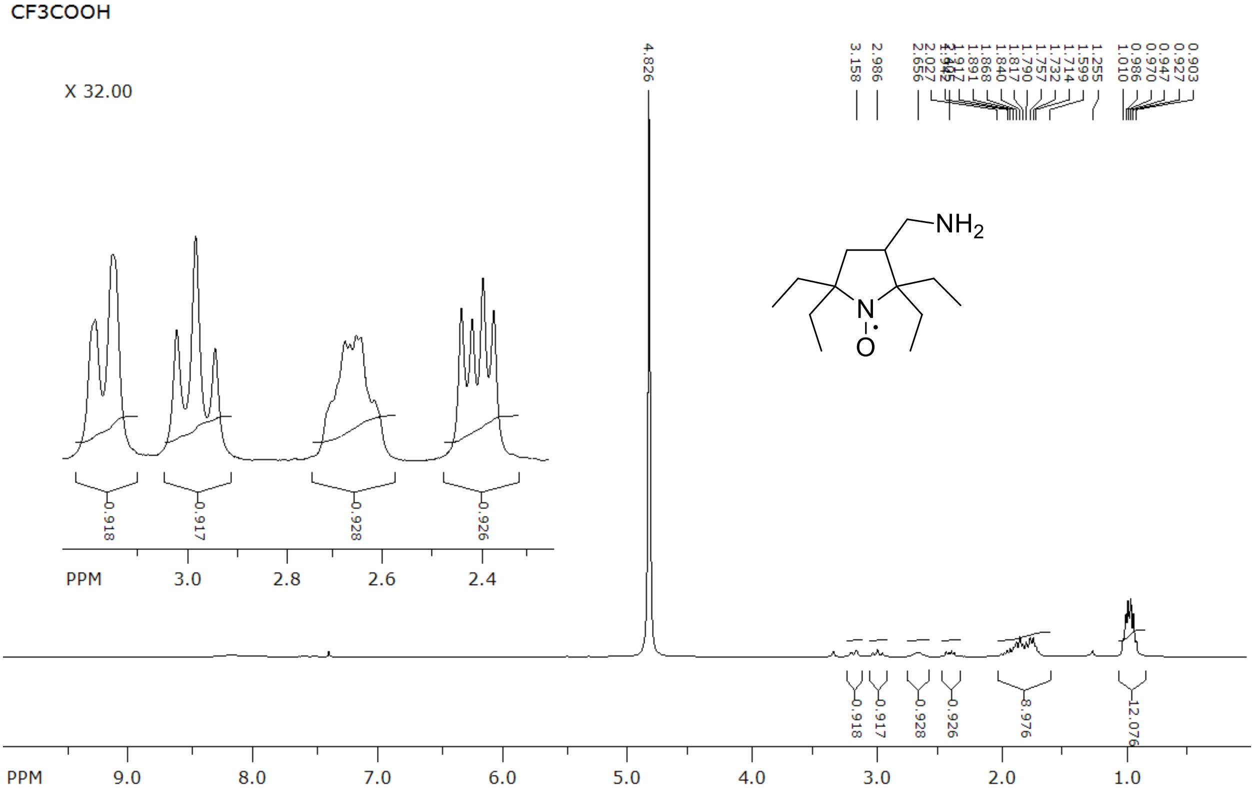

^{1}H NMR (300 MHz; CD3OD/CDCl3, Zn/CF3COOH) of 3-(2-triphenylphosphonio-acetamido)methyl-2,2,5,5-tetraethylpyrrolidine-1-oxyl chloride (28).

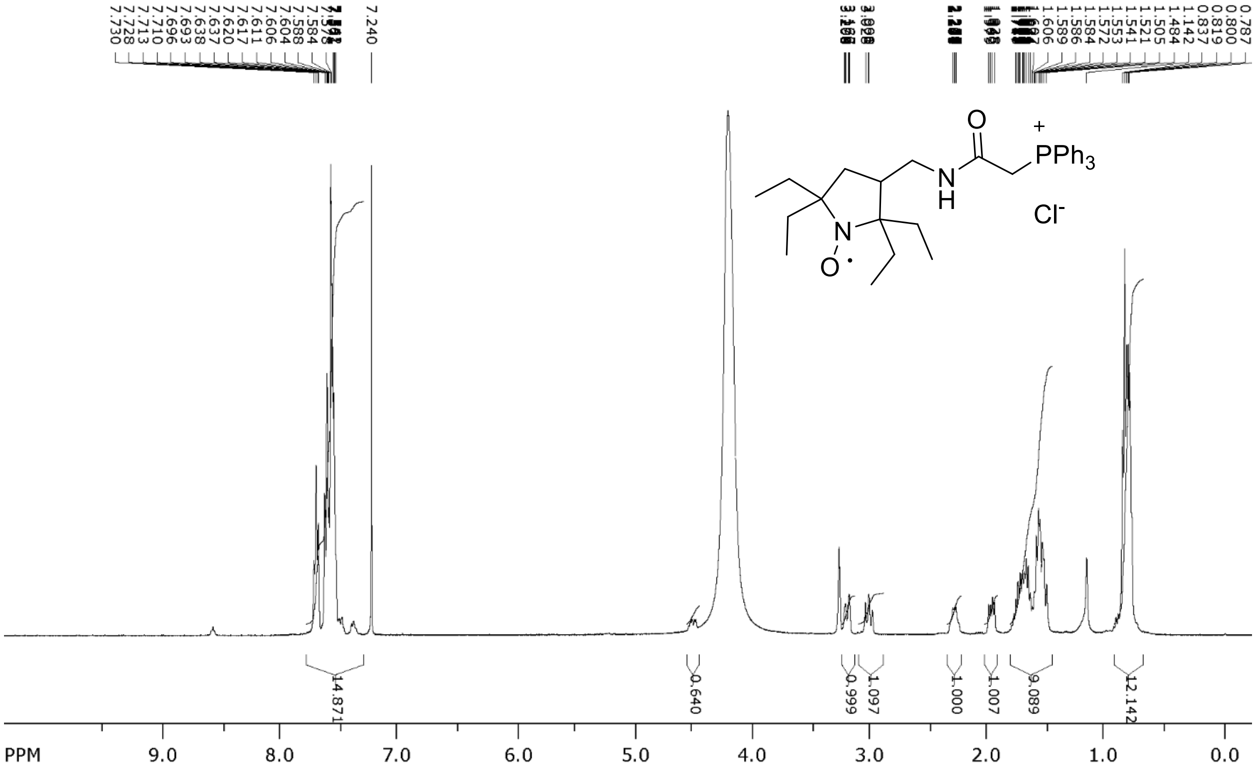

^{1}HNMR (300 MHz; $CD_3OD/CDCl_3$, Zn/CF_3COOH) of 3-(2-(pyrrolidin-1yl)acetamidomethyl)-2,2,5,5-tetraethylpyrrolidine-1-oxyl (29).

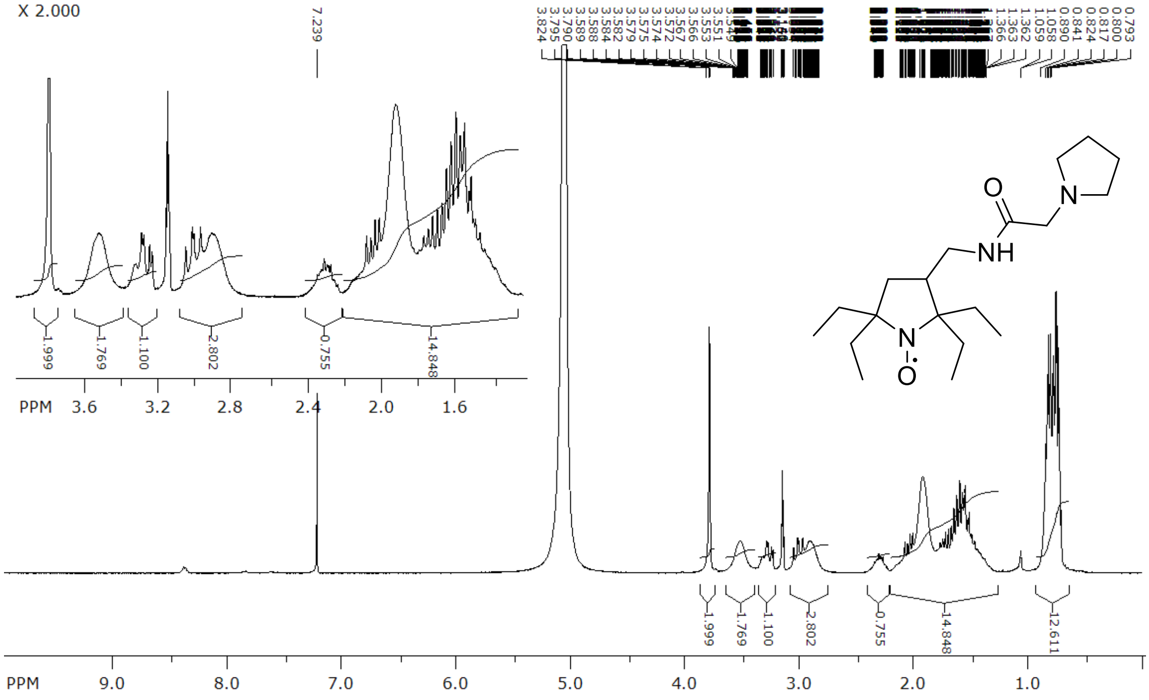

^{1}HNMR (300 MHz; $CD_3OD/CDCl_3$, Zn/CF_3COOH) of 3-((piperidin-1-yl)methyl)-2,2,5,5-tetraethylpyrrolidine-1-oxyl (30).

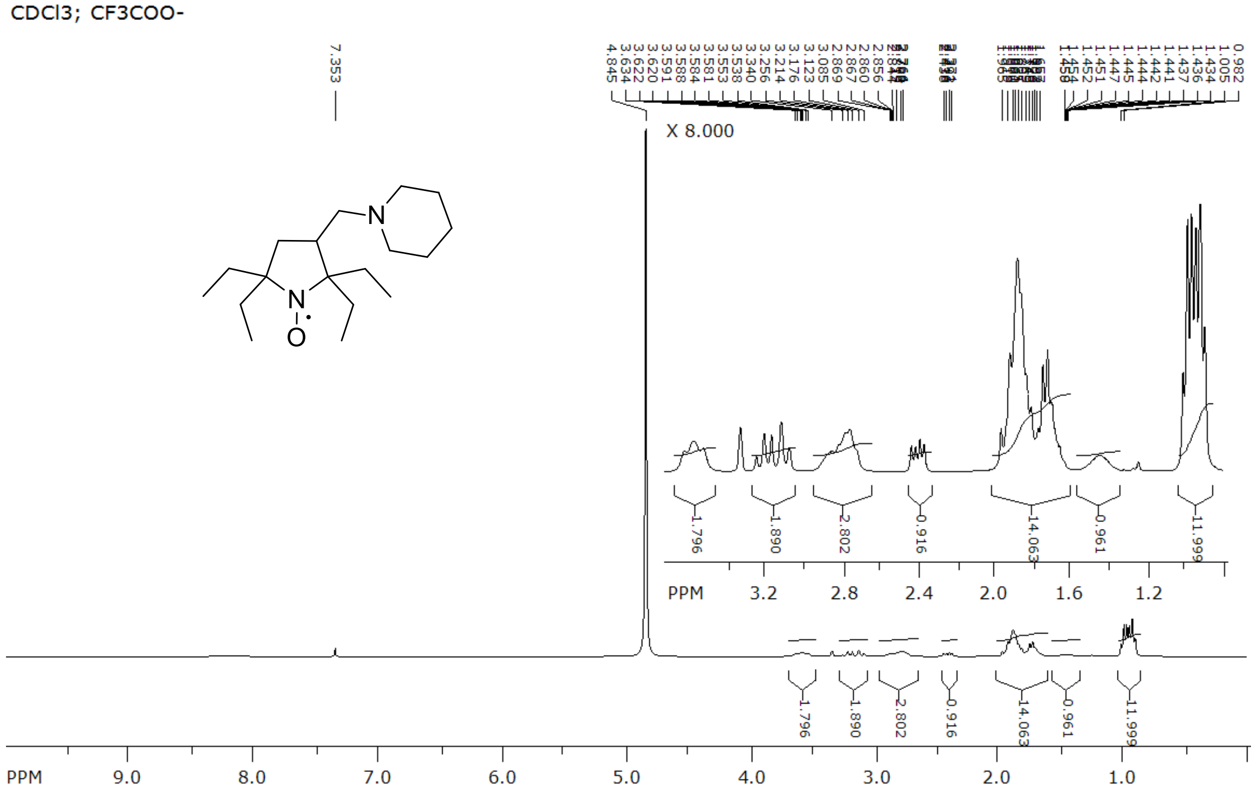

^{1}H NMR(300 MHz; $CD_3OD/CDCl_3$, Zn/CF_3COOH) of 3-(dimethylaminomethyl)-2,2,5,5-tetraethylpyrrolidine-1-oxyl (31).

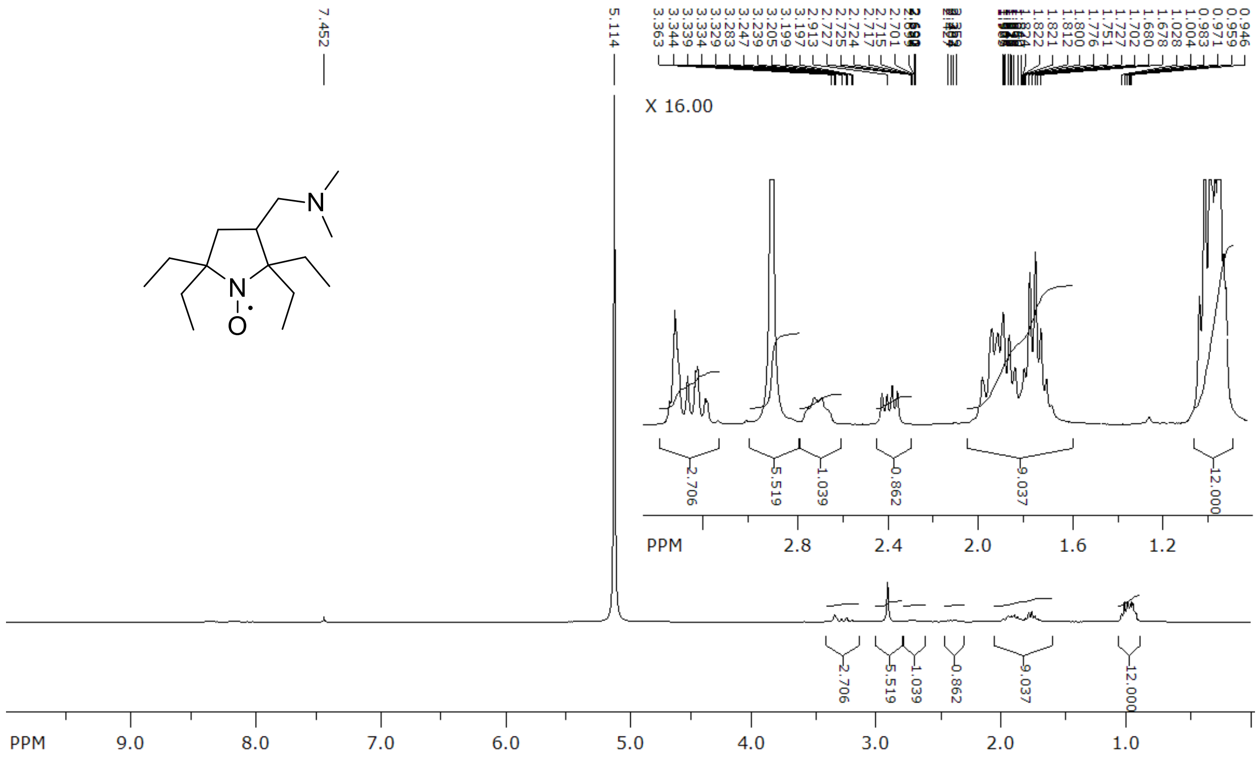

Line shape analysis of multiplets for mixture of isomers 10a,b.

The analysis was performed using the gNMR 5.0 software is shown in Fig. S1. Parameters of spin system are shown in Table S1 [Budzelaar, P. H. M. "gNMR, version 5.0. 6.0." *Ivorysoft, Nijmegen, Netherlands* (2006).]

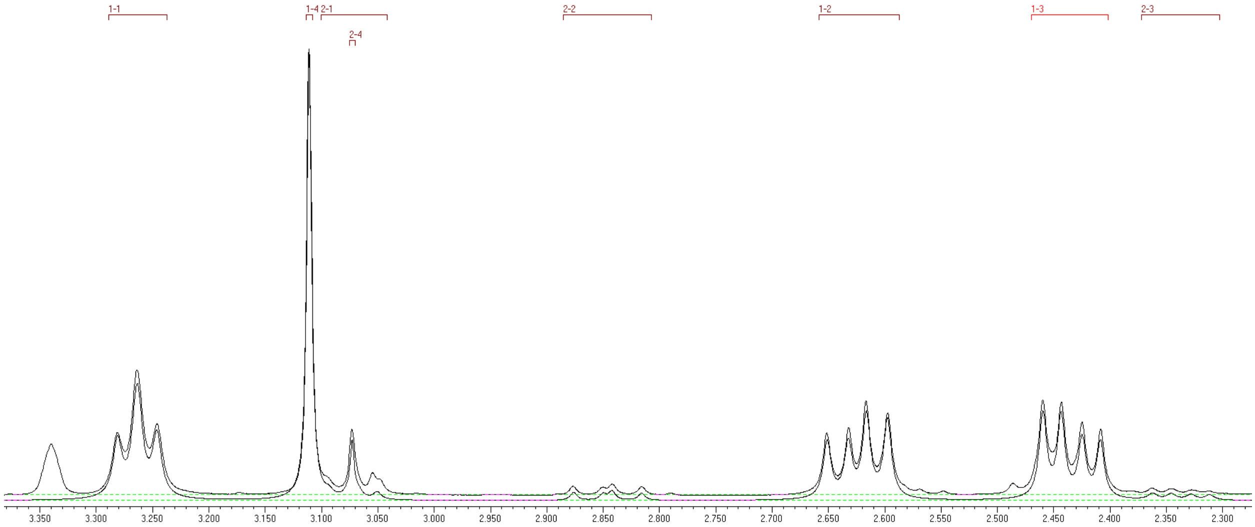

Fig. S1 Simulation of spin system for ^1H NMR (400 MHz; $CD_3OD/CDCl_3$, Zn/CF_3COOH system) of 3-carboxy-5-ethinyl-2,2,5-triethylpyrrolidine-1-oxyl – mixture of isomers (10a,b).

Table S1.Simulation of spin system parameters for ^1H NMR (400 MHz; $CD_3OD/CDCl_3$, Zn/CF_3COOH system) of 3-carboxy-5-ethinyl-2,2,5-triethylpyrrolidine-1-oxyl – mixture of isomers (10a,b).

Major isomer – 10a							
#	Nucleus	n	Shift	J[1]	J[2]	J[3]	J[4]
1	^1H	1	3.263	-			
2	^1H	1	2.623	7.55			
3	^1H	1	2.436	6.49	-13.94		
4	^1H	1	3.111	-	-	-	
Concentration: 86.87975							
Minor isomer -10b							
#	Nucleus	n	Shift	J[1]	J[2]	J[3]	J[4]
1	^1H	1	3.071				
2	^1H	1	2.846	10.51			
3	^1H	1	2.337	6.65	-13.72		
4	^1H	1	3.073	-	-	-	-
Concentration: 8.25143							

IR spectra

IR (KBr) of 3-carboxy-2,2,5,5-tetraethylpyrrolidine-1-oxyl (1).

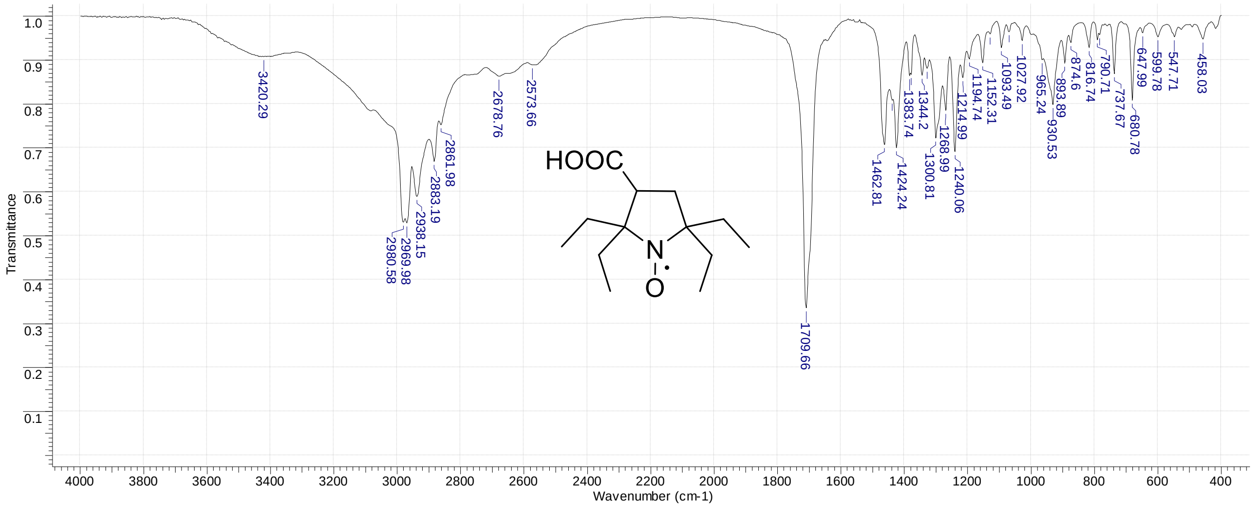

IR (neat) of 3-[[(2,5-dioxo-1-pyrrolidinyl)oxy]carbonyl]-2,2,5,5-tetraethylpyrrolidine-1-oxyl (3).

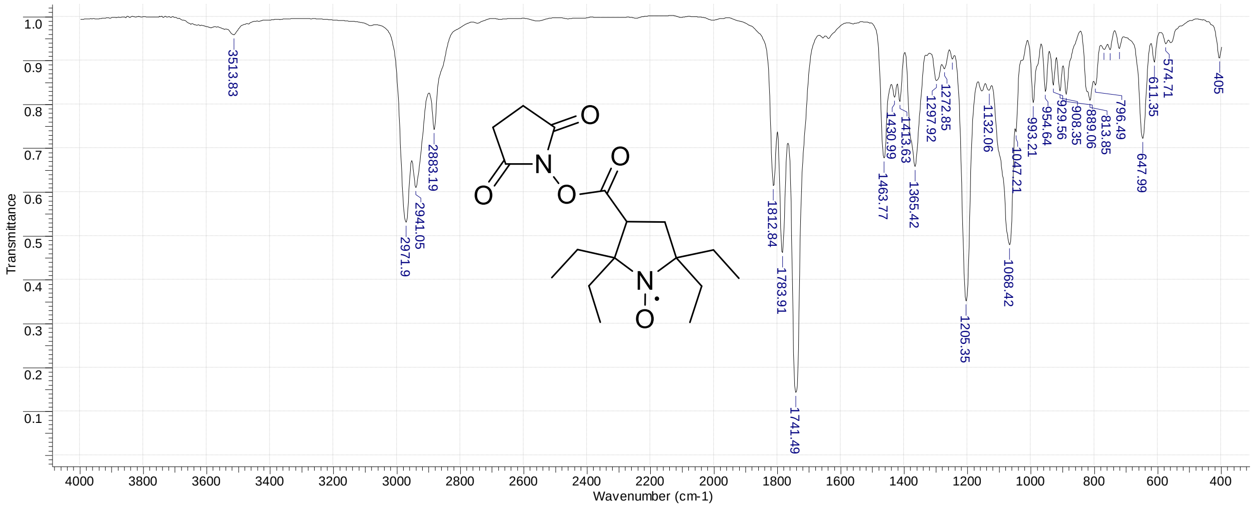

IR (KBr) of 3-carboxy-2,2,5-triethyl-3,4-dihydro-2H-pyrrole 1-oxide (8).

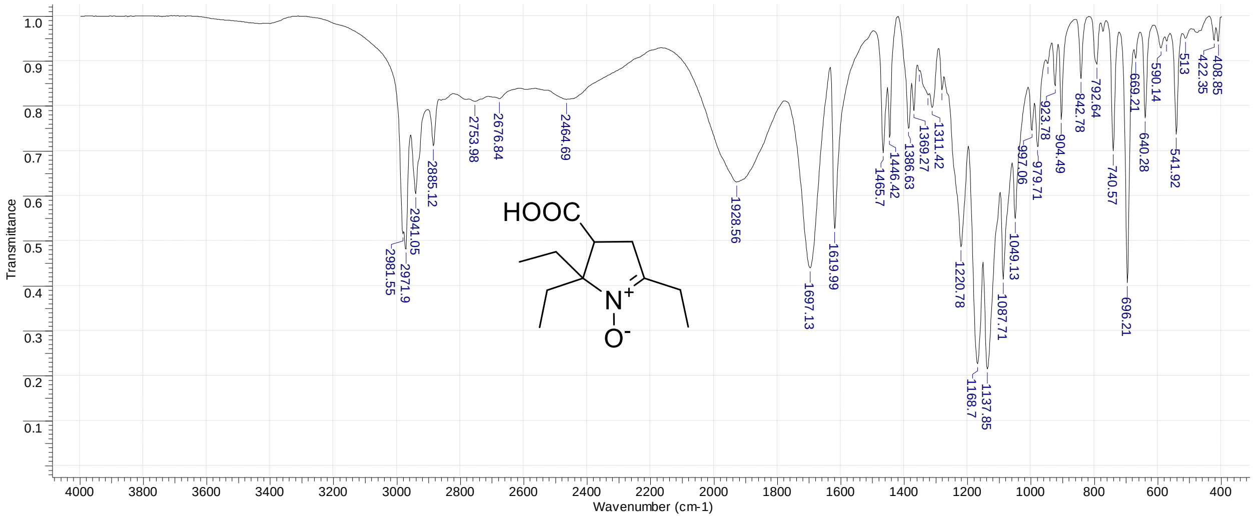

IR (KBr) of 3-carboxy-5-ethynyl-2,2,5-triethylpyrrolidine-1-oxyl (10a,b).

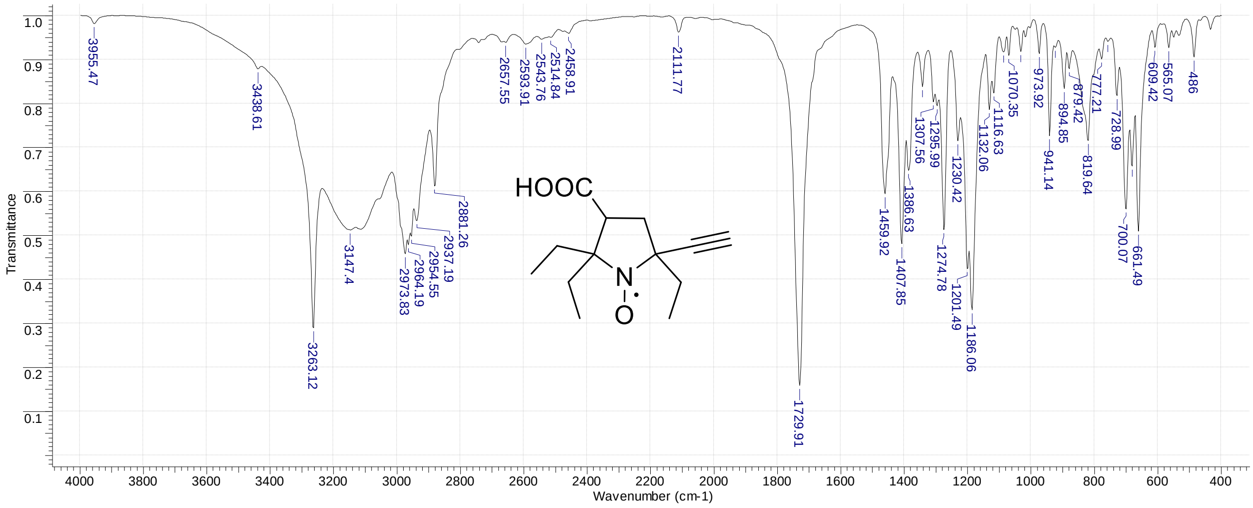

IR (KBr) of 3-carboxy-5-vinyl-2,2,5-triethylpyrrolidine-1-oxyl (14).

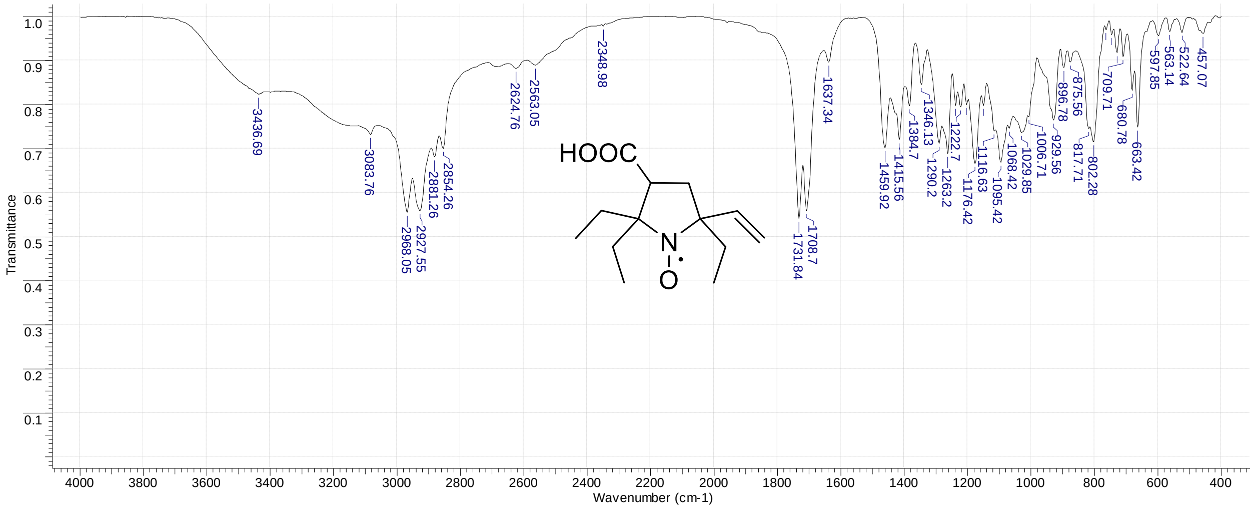

IR (KBr) of 3-carboxy-2,2,5,5-tetraethylpyrrolidine (15).

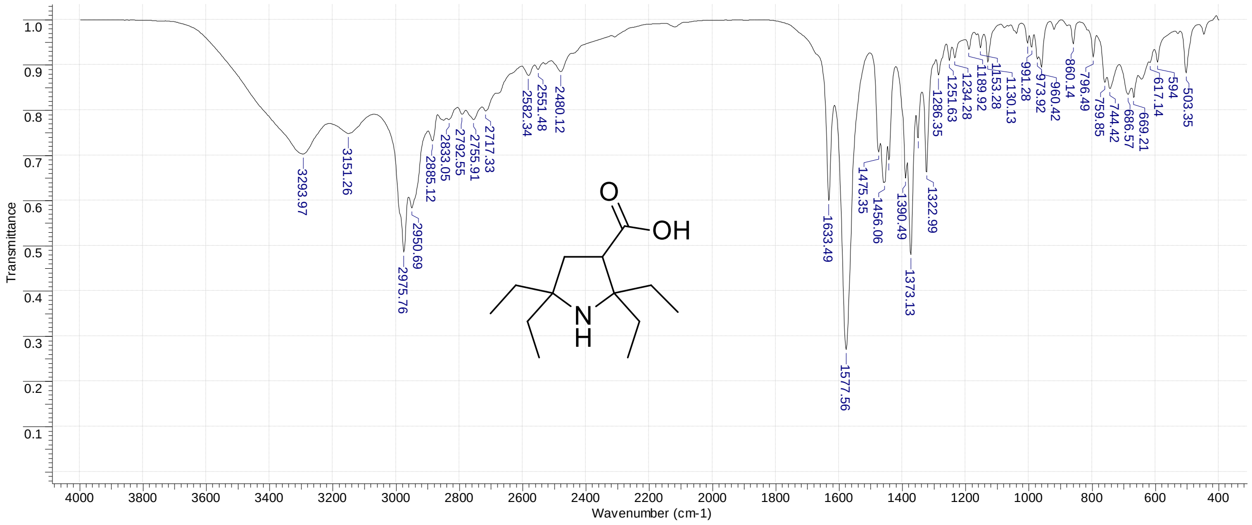

IR (neat) of 3-methoxycarbonyl-2,2,5,5-tetraethylpyrrolidine-1-oxyl (16).

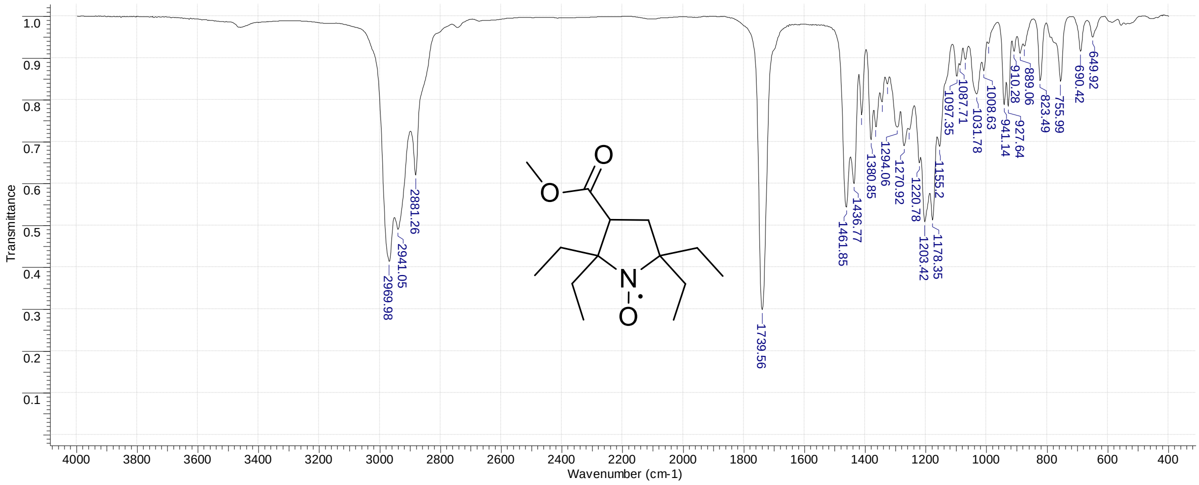

IR (KBr) of 1-hydroxy-3-methoxycarbonyl-2,2,5,5-tetraethylpyrrolidine hydrochloride (17)

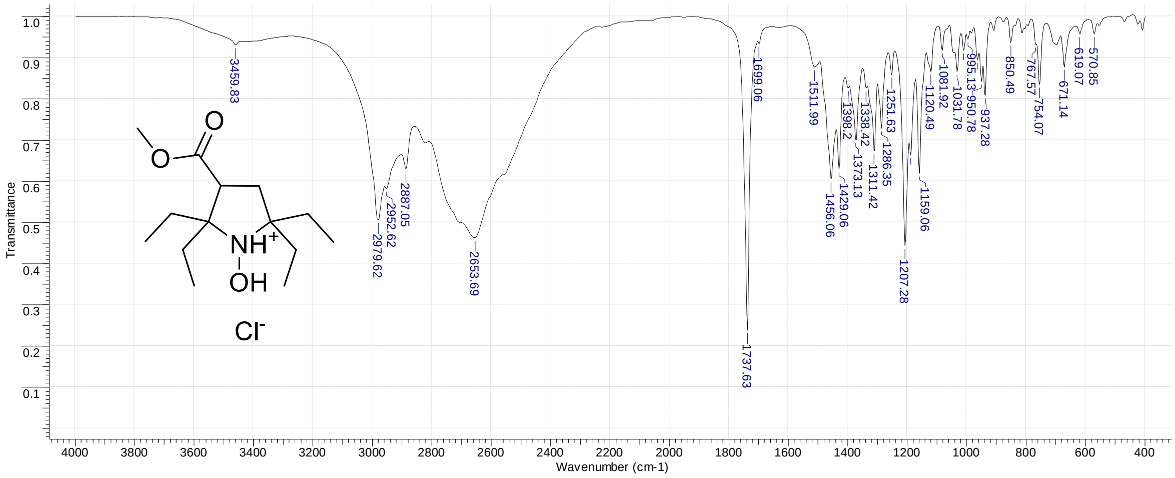

IR (neat) of 3-hydroxymethyl-2,2,5,5-tetraethylpyrrolidine-1-oxyl (18).

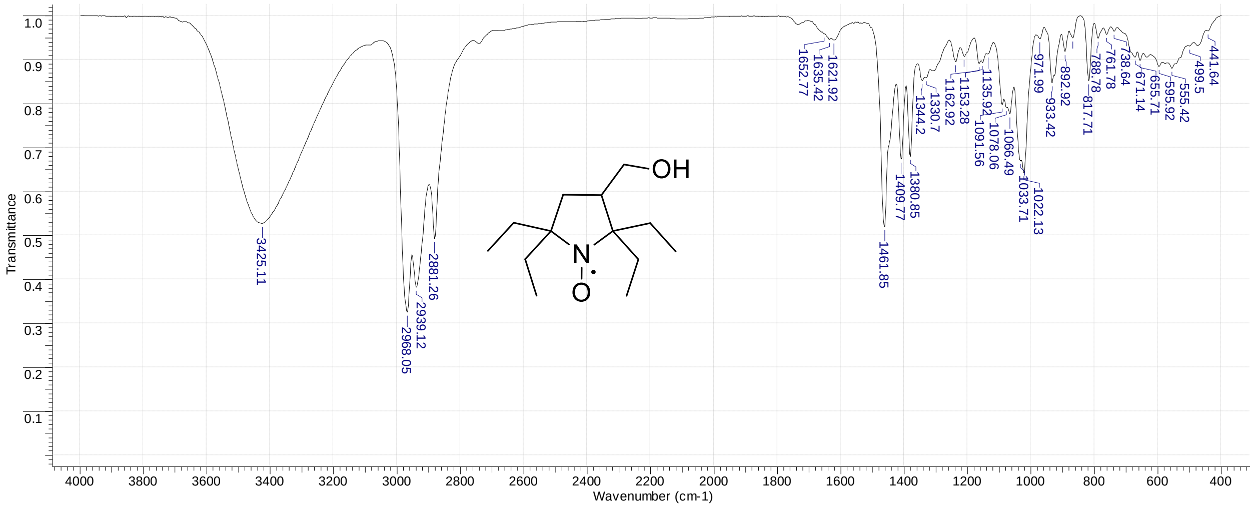

IR (neat) of 3-hydroxymethyl-5-ethinyl-2,2,5-triethylpyrrolidine-1-oxyl (19a,b).

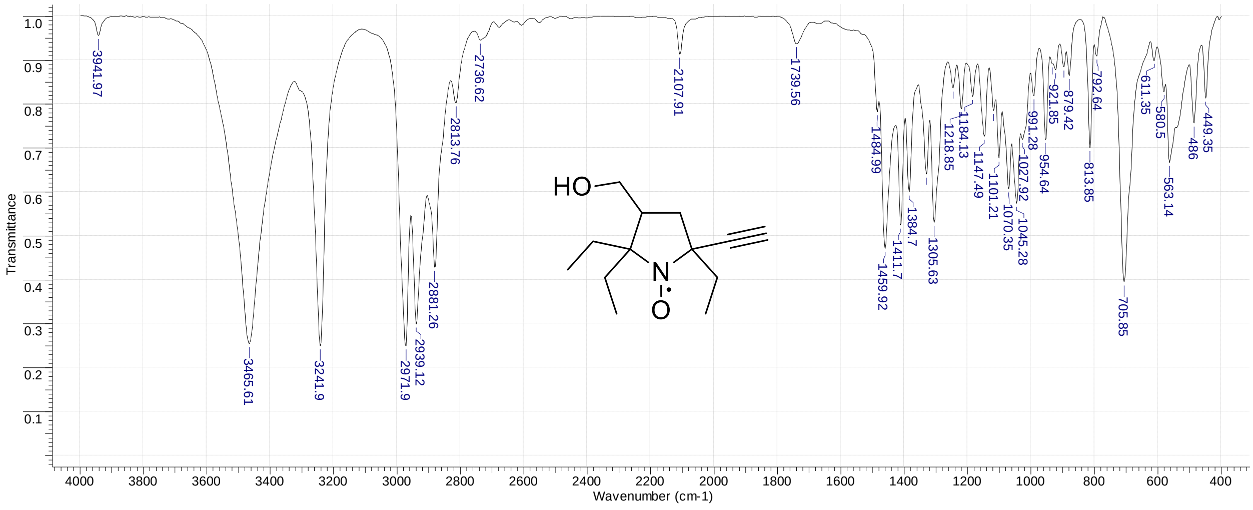

IR (neat) of 3-brommethyl-2,2,5,5-tetraethylpyrrolidine-1-oxyl (20a).

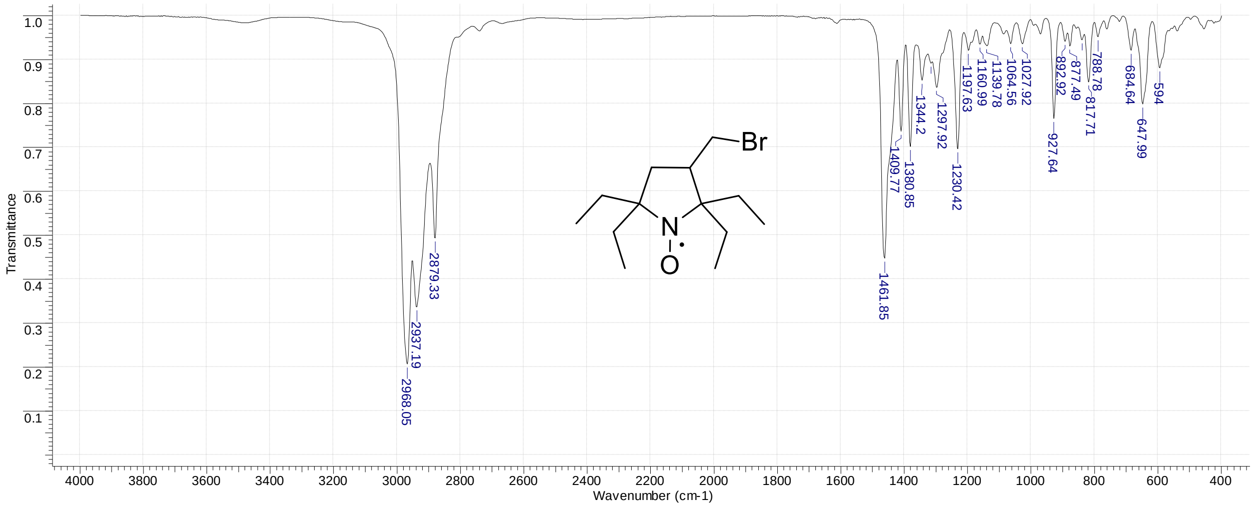

IR (neat) of 3-mesyloxymethyl-2,2,5,5-tetraethylpyrrolidine-1-oxyl (20b).

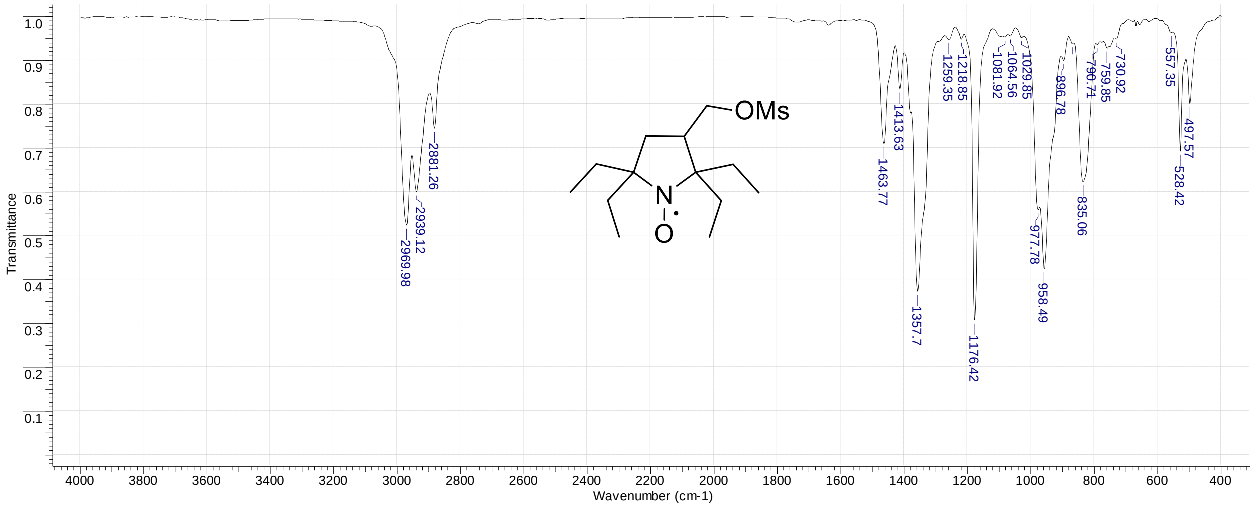

IR (neat) of 3-azidomethyl-2,2,5,5-tetraethylpyrrolidine-1-oxyl (21).

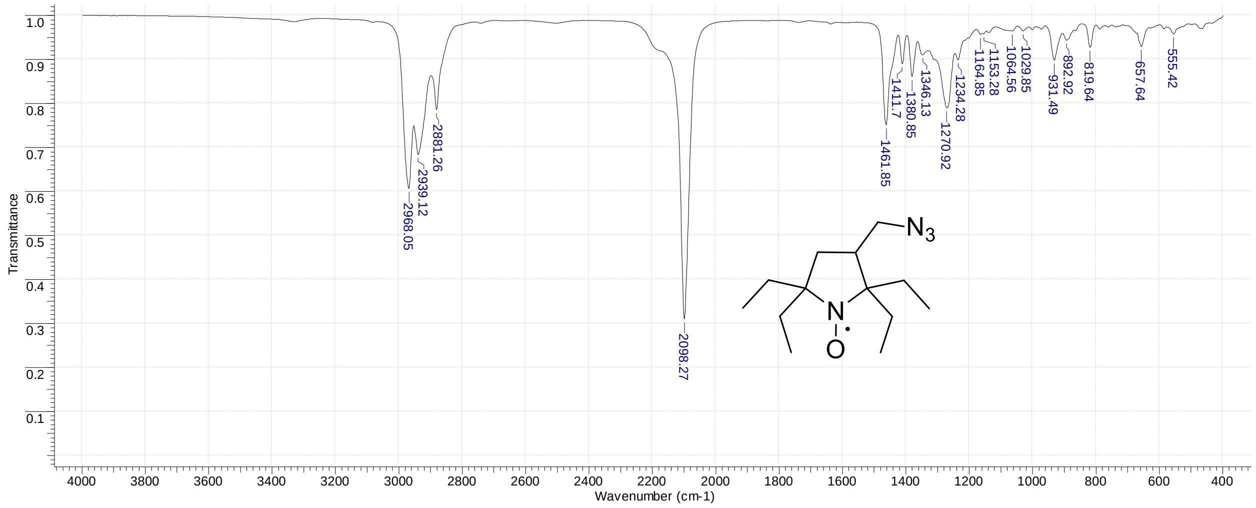

IR (neat) of 3-aminomethyl-2,2,5,5-tetraethylpyrrolidine-1-oxyl (22).

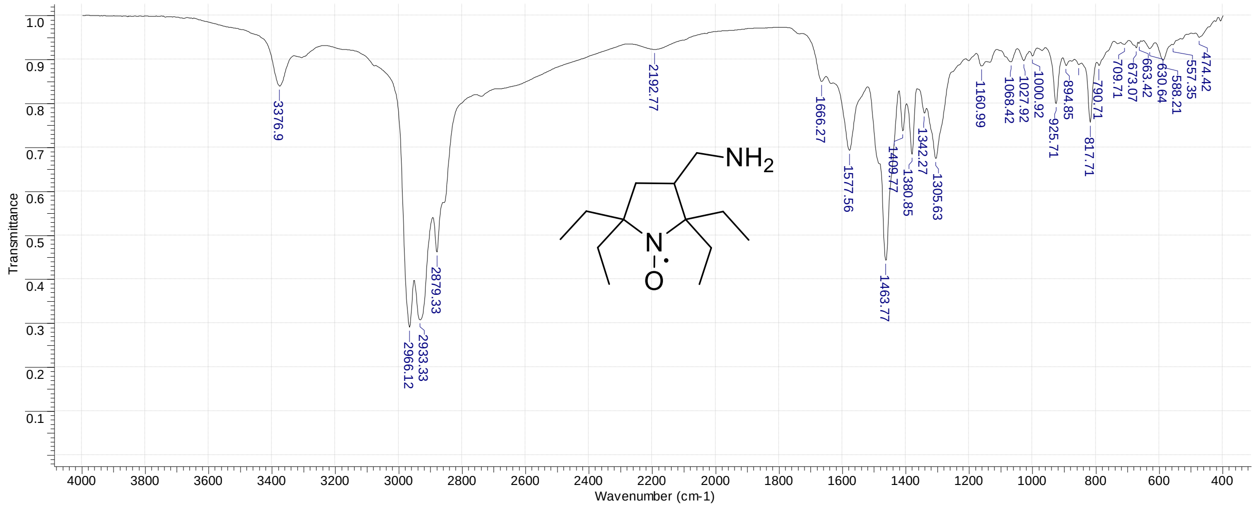

IR (KBr) of 3-maleimidomethyl-2,2,5,5-tetraethylpyrrolidine-1-oxyl (24).

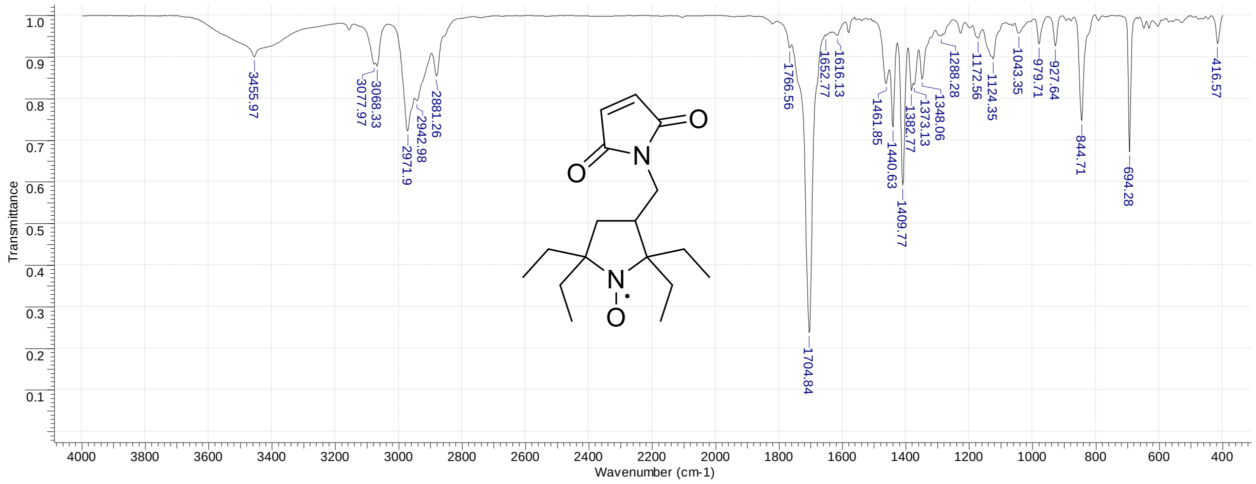

IR (neat) of 3-bromoacetamidomethyl-2,2,5,5-tetraethylpyrrolidine-1-oxyl (25).

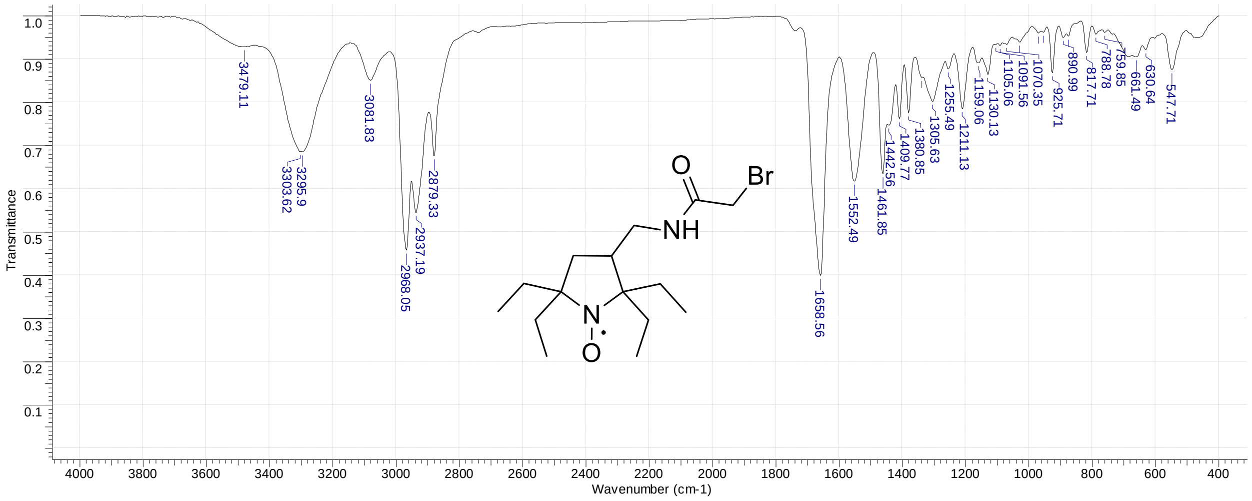

IR (neat) of 3-Chloroacetamidomethyl-2,2,5,5-tetraethylpyrrolidine-1-oxyl (26).

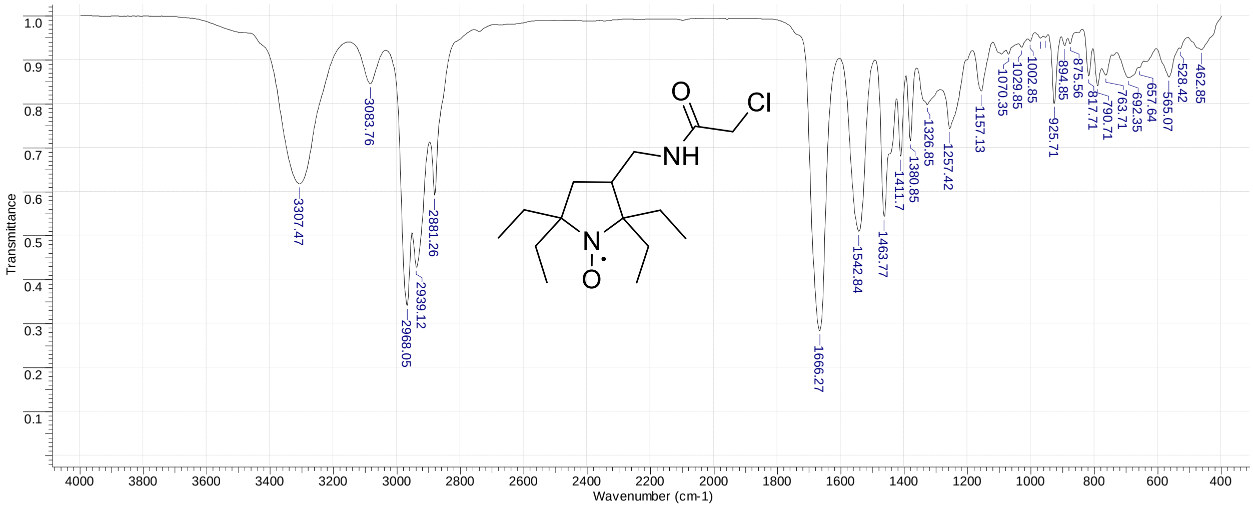

IR (KBr) of 3-Iodoacetamidomethyl-2,2,5,5-tetraethylpyrrolidine-1-oxyl (27).

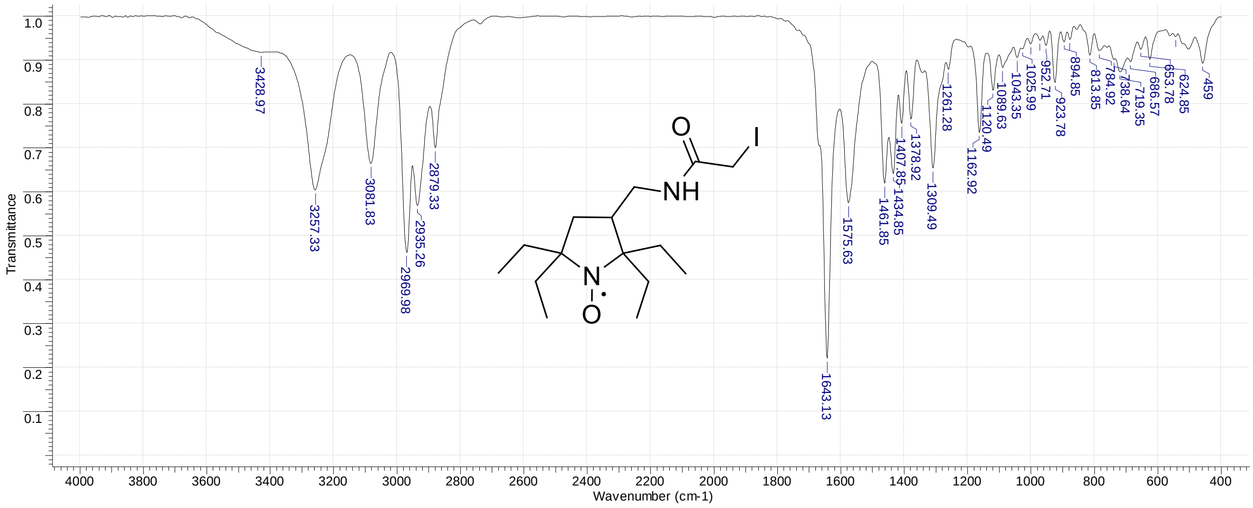

IR (KBr) of 3-(2-triphenylphosphonio-acetamido)methyl-2,2,5,5-tetraethylpyrrolidine-1-oxyl chloride (28).

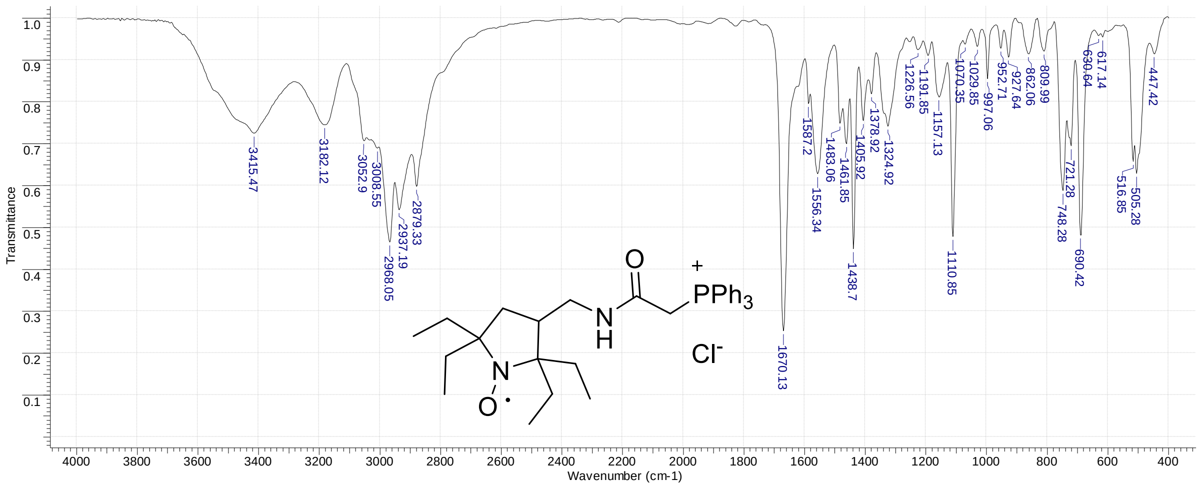

IR (neat) of 3-(2-(pyrrolidin-1yl)acetamidomethyl)-2,2,5,5-tetraethylpyrrolidine-1-oxyl (29).

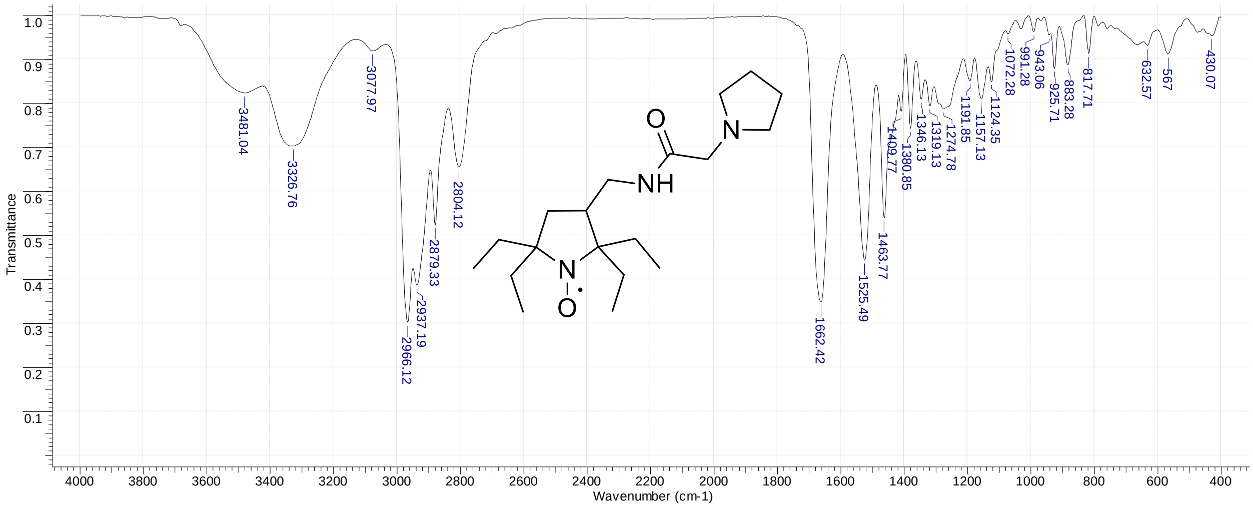

IR (neat) of 3-((piperidin-1-yl)methyl)-2,2,5,5-tetraethylpyrrolidine-1-oxyl (30).

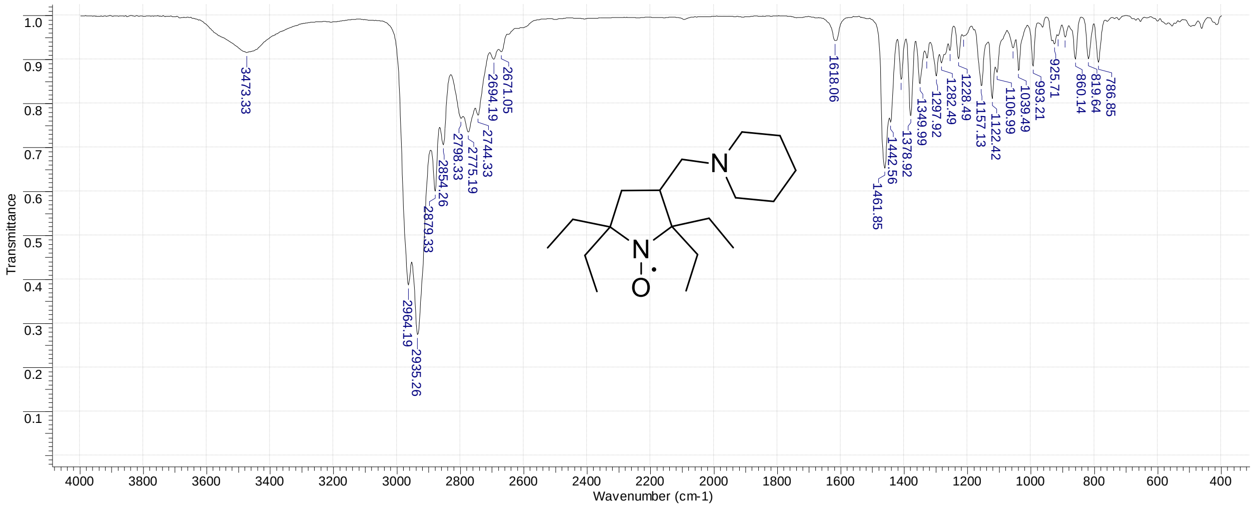

IR (neat) of 3-(dimethylaminomethyl)-2,2,5,5-tetraethylpyrrolidine-1-oxyl (31).

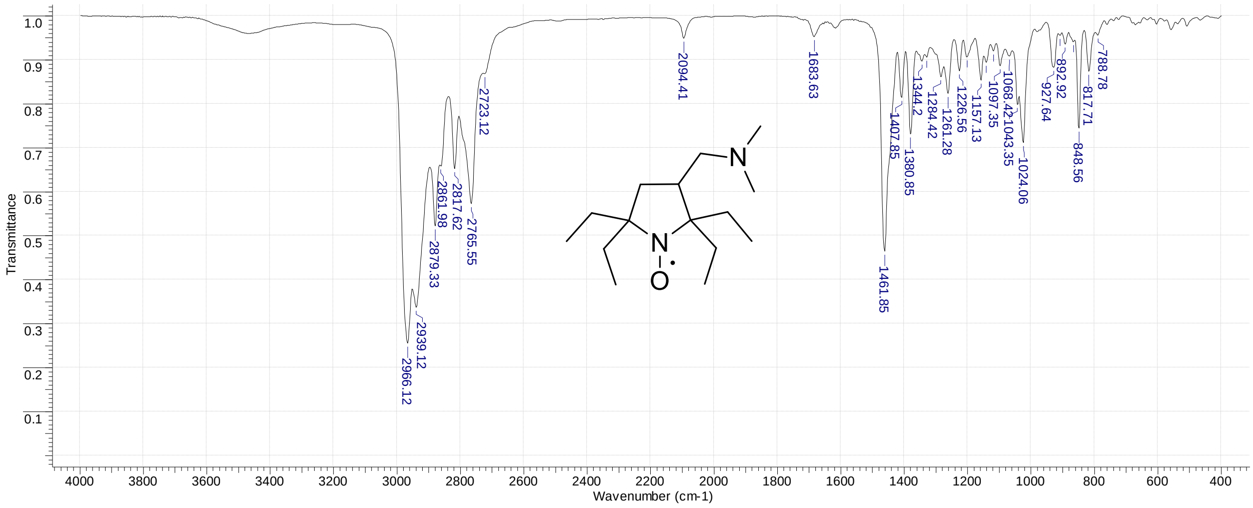

HPLC analyses

HPLC of 3-carboxy-5-ethinyl-2,2,5-triethylpyrrolidine-1-oxyl major isomer(19a).

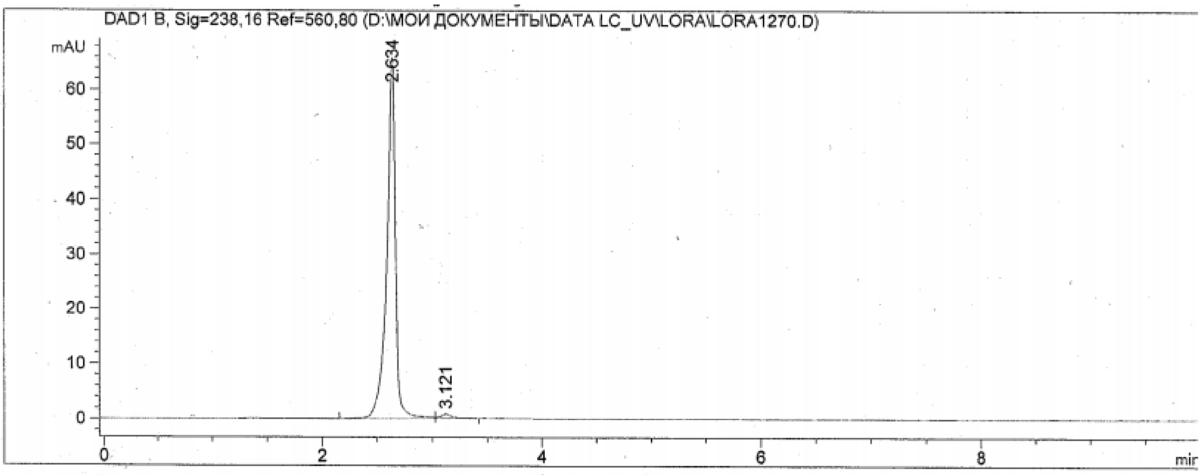

Area Percent Report

```
Sorted By            :      Signal
Multiplier:                 :        1.0000
Dilution:                   :        1.0000
Use Multiplier & Dilution Factor with ISTDs
```

Signal 1: DAD1 B, Sig=238,16 Ref=560,80

Peak #	RetTime [min]	Type	Width [min]	Area [mAU*s]	Height [mAU]	Area %
1	2.634	BV	0.0767	354.74719	66.02786	98.3314
2	3.121	VB	0.1055	6.01980	8.02957e-1	1.6686
Totals :				360.76699	66.83082	

HPLC of 3-((piperidin-1-yl)methyl)-2,2,5,5-tetraethylpyrrolidine-1-oxyl (30).

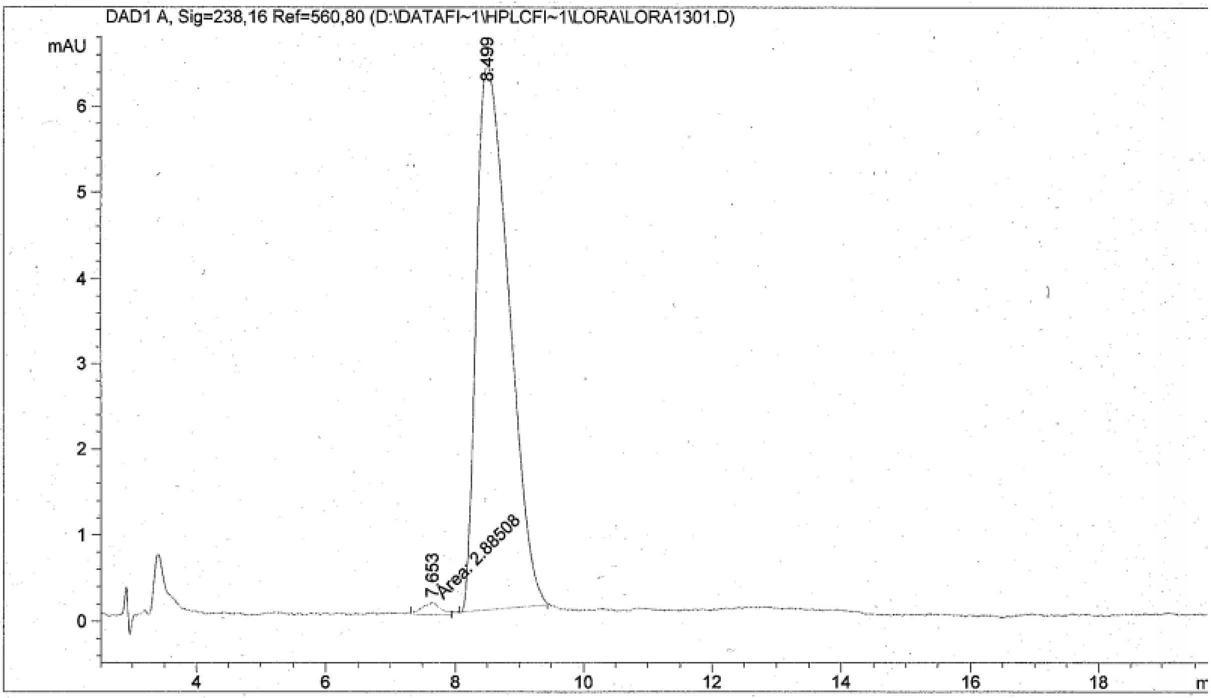

Area Percent Report

Sorted By : Signal
Multiplier : 1.0000
Dilution : 1.0000
Use Multiplier & Dilution Factor with ISTDs

Signal 1: DAD1 A, Sig=238,16 Ref=560,80

Peak #	RetTime [min]	Type	Width [min]	Area [mAU*s]	Height [mAU]	Area %
1	7.653	MM	0.3434	2.88508	1.40031e-1	1.2723
2	8.499	BB	0.5095	223.88176	6.34769	98.7277
Totals :				226.76684	6.48772	

HPLC of 3-(2-(pyrrolidin-1yl)acetamidomethyl)-2,2,5,5-tetraethylpyrrolidine-1-oxyl (29).

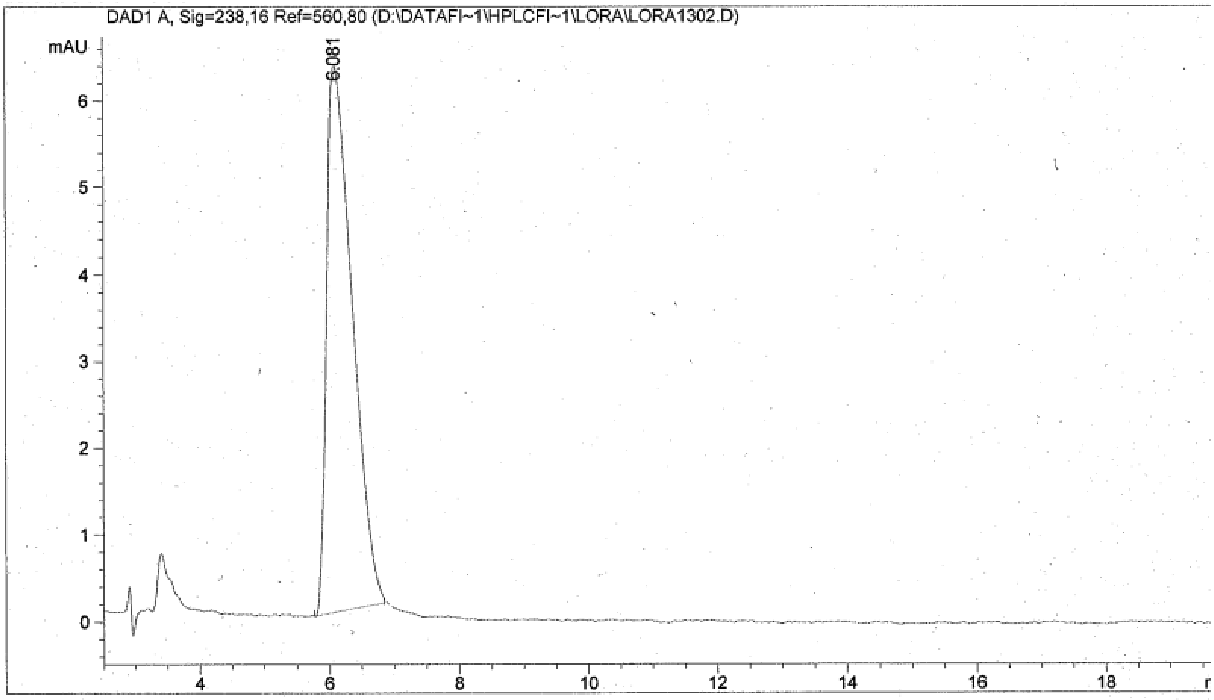

Area Percent Report

Sorted By : Signal
Multiplier : 1.0000
Dilution : 1.0000
Use Multiplier & Dilution Factor with ISTDs

Signal 1: DAD1 A, Sig=238,16 Ref=560,80

Peak #	RetTime [min]	Type	Width [min]	Area [mAU*s]	Height [mAU]	Area %
1	6.081	PB	0.3941	172.00441	6.30903	100.0000
Totals :				172.00441	6.30903	

Table S2. X-ray experimental details

**Crystal data**	Compound **10a** (dsa112k)
Chemical formula	$C_{13}H_{20}NO_3$
M_r	238.30
Crystal system, space group	Orthorhombic, $Pna2_1$
Temperature (K)	296
a, b, c (Å)	19.2686 (9), 8.1112 (3), 8.6742 (3)
V (Å^3)	1355.70 (9)
Z	4
Radiation type	Mo $K\alpha$
μ (mm^{-1})	0.08
Crystal size (mm)	0.61 × 0.32 × 0.12
**Data collection**	
Diffractometer	Bruker *APEX*-II CCD
Absorption correction	Multi-scan<br>*SADABS2008*/1
T_{min}, T_{max}	0.911, 0.956
No. of measured, independent and observed [$I > 2\sigma(I)$] reflections	16081, 3131, 2834
R_{int}	0.044
(sin $\theta/\lambda)_{max}$ (Å^{-1})	0.659
**Refinement**	
$R[F^2 > 2\sigma(F^2)]$, $wR(F^2)$, S	0.040, 0.113, 1.03
No. of reflections	3131
No. of parameters	160
No. of restraints	2
H-atom treatment	H atoms treated by a mixture of independent and constrained refinement
$\sigma\rangle_{max}$, $\sigma\rangle_{min}$ (e Å^{-3})	0.34, -0.17
Absolute structure parameter	0.2 (3)
CCDC number	2111214

Fig.S2. X-Ray of 3-carboxy-5-ethynyl-2,2,5-triethylpyrrolidine-1-oxyl (10a).

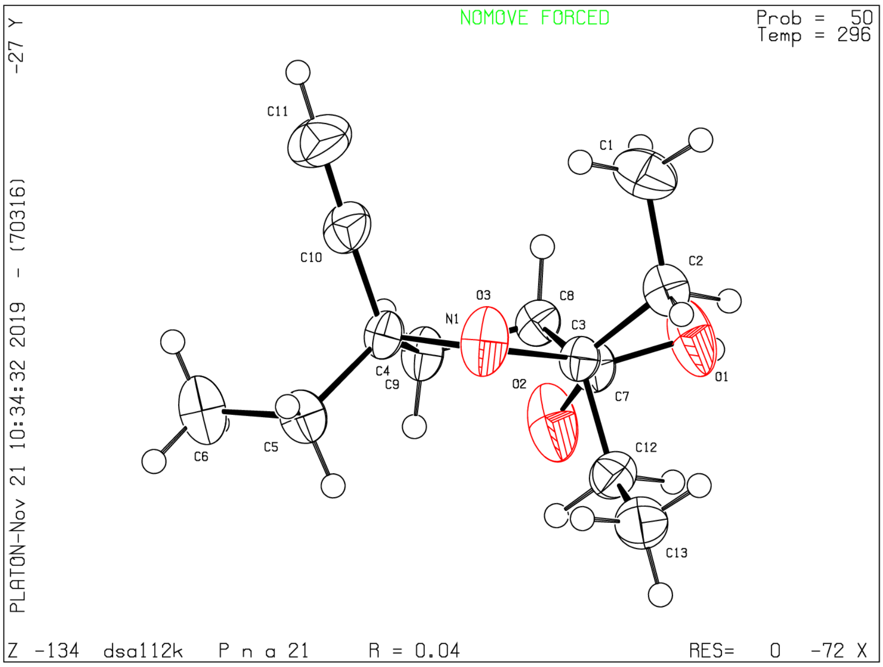

Computer programs: Bruker *APEX2*, Bruker *SAINT*, *SHELXS2018* (Sheldrick, 2018), *SHELXL2018*/3 (Sheldrick, 2018), Bruker *SHELXTL*.

Fig. S3. The dependences of the first order rates of radical reductions versus ascorbic acid concentrations.

Data were fitted by linear dependences starting at zero. Second order reaction rate constant is a slope of such dependence.

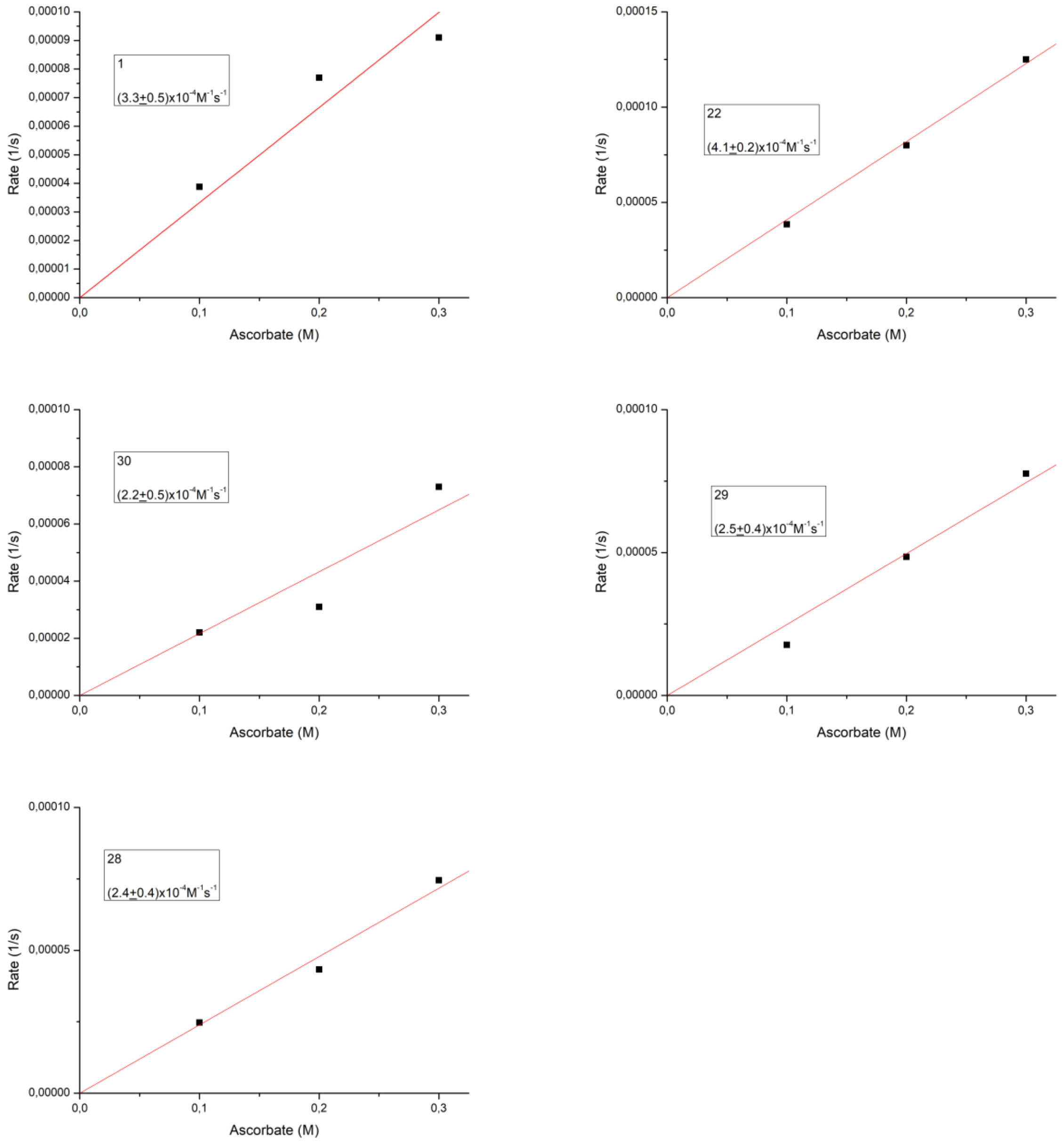

Supplement: Supplementary file 1 [file molecules-26-05761-s001.zip › molecules-1384564 SF.pdf]
